# Bio-based poly(benzimidazole-*co*-amide)-derived N, O co-doped carbons as fast-charging anodes for lithium-ion batteries†

**DOI:** 10.1039/d4na00416g

**Published:** 2024-08-27

**Authors:** Kottisa Sumala Patnaik, Bharat Srimitra Mantripragada, Rajashekar Badam, Koichi Higashimine, Xianzhu Zhong, Tatsuo Kaneko, Noriyoshi Matsumi

**Affiliations:** a Graduate School of Advanced Science and Technology, Japan Advanced Institute of Science and Technology (JAIST) Nomi Ishikawa 923-1292 Japan matsumi@jaist.ac.jp; b Centre for Nano Materials and Technology, Japan Advanced Institute of Science and Technology (JAIST) Nomi Ishikawa 923-1292 Japan; c Division of Transdisciplinary Sciences, Japan Advanced Institute of Science and Technology (JAIST) Nomi Ishikawa 923-1292 Japan; d Elements Strategy Initiative for Catalysts and Batteries (ESCIB), Kyoto University Nishikyo-ku Kyoto 615-8245 Japan

## Abstract

Lithium-ion batteries (LIBs) that can be charged faster while delivering high capacity are currently in significant demand, especially for electric vehicle applications. In this context, this study introduces a less-explored subject: nitrogen and oxygen dual-doped carbons derived from bio-based copolymers, specifically poly(benzimidazole-*co*-amide). The synthesis involved varying proportions of benzimidazole to amide, namely, 8.5 : 1.5, 7 : 3, and 5 : 5. The copolymers were pyrolyzed under a nitrogen atmosphere to obtain co-doped carbons, wherein the copolymers acted as single sources of carbon, nitrogen, and oxygen, with the nitrogen content ranging between 12.1 and 8.0 at% and oxygen doping between 11.8 and 25.0 at%, and were named as pyrolyzed polybenzimidazole-*co*-amide 8.5–1.5, 7–3, and 5–5. Coin cells were fabricated and rate studies were conducted for all three samples, wherein PYPBIPA8.5–1.5 outperformed all others, especially at higher current densities. Intrigued by these interesting results, when long-cycling studies were performed at a high current density of 4.0 A g^−1^, pyrolysed polybenzimidazole-*co*-amide 8.5–1.5 showed a delithiation capacity of 135 mA h g^−1^ compared to pyrolysed polybenzimidazole-*co*-amide 7–3 and 5–5 with a delithiation capacity of 100 mA h g^−1^ and 60 mA h g^−1^, respectively, with a capacity retention of 90% even after 3000 cycles. Furthermore, a full cell (2025-coin cell) was fabricated using the PYPBIPA8.5–1.5 anode and LiNi_0.80_ Co_0.15_Al_0.05_O_2_ (LiNCAO) cathode.

## Abstract 2

This works presents a nitrogen and oxygen co-doped carbon obtained from a bio-based polymer as anode active material for fast charging in lithium-ion batteries.

## Graphical abstract

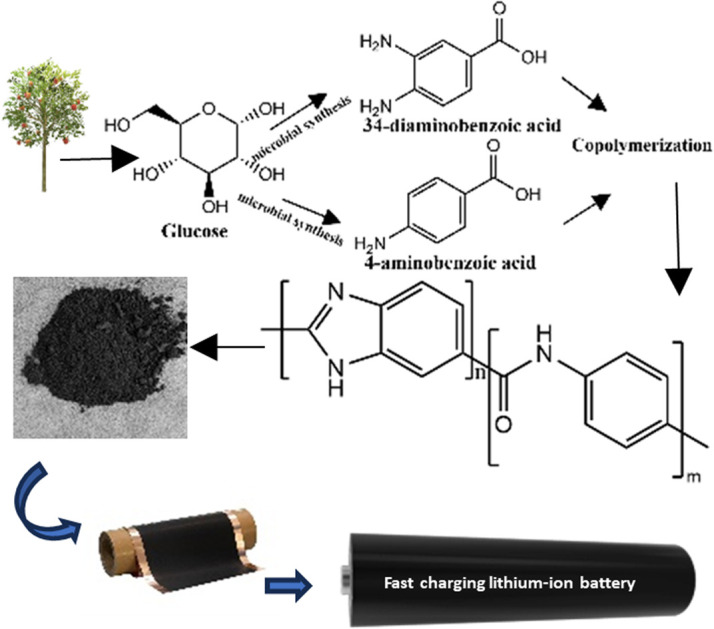

## Introduction

Ever since LIBs were first discovered in the 1990s, their applications have expanded tremendously.^1^ In particular, the recent past has witnessed the implementation of LIBs in diverse devices, ranging from earphones to electric vehicles (EVs). EVs are currently the most environmental friendly means of transport available in the market. However, the state-of-the-art EVs are still unable to completely satisfy customer demands with respect to their driving range and the time needed for charging. Batteries that can be charged in less time afford only a few miles, whereas batteries that can sustain a long distance take more time for charging, thus resulting in a trade-off between long distance operation and fast charging.^2–4^ To overcome these problems, various types of anode materials are currently being investigated across the world.^5,6^ Of all the tested materials, carbonaceous materials seem to be the best choice owing to their non-toxicity, low cost, and simplicity in synthetic procedures.^7,8^ Among the various carbon-based materials, graphite seems to be one of the most preferred anode materials; however, its slow Li-ion kinetics inside the bulk material limits its fast-charging applications.^9,10^ Hence, there is an urgent need to discover novel carbon materials that can afford faster Li-ion kinetics. In light of this, hard carbons (HCs) seem to be an excellent choice as anode materials for fast charging. HCs are materials with short-range graphitized domains arranged in a disordered manner, with a large number of nanovoids and defect sites.^11^ Unlike graphite, owing to their unique structure, they can store Li-ions not only through intercalation^12^ between carbon planes but also through surface adsorption at defect sites, edges, and nanovoids, thus contributing to faster Li-ion kinetics. Alongside, heteroatom doping using elements such as N, O, S, or P, can contribute to faster kinetics owing to the occurrence of surface-based redox reactions between heteroatoms and Li ions.^12,13^ The introduction of heteroatoms can also induce defect sites that promote the perpendicular movement of ions from one plane to another plane, boost electrical conductivity and reduce charge-transfer impedance, which further benefit fast charging.^14,15^ Hence, heteroatom-doped hard carbons having the combined benefits of both counterparts could be a perfect choice for carbon-based anode materials for achieving fast-charging batteries. Previously, our group reported a heavily nitrogen-doped hard carbon endowed with a nitrogen doping of approx. 17 wt% as an extremely fast-charging anode for LIBs. Due to the synergistic effects of heavy nitrogen doping using a polymer source, large number of defect sites, enlarged *d*-spacing, the LIB exhibited an extremely fast-charging (XFC) capability at 18.6 A g^−1^ and ultralong cyclability of 3000 cycles with 90% capacity retention.^16^ These results encouraged our group to further explore the effect of dual heteroatom doping in hard carbons for fast-charging applications. Though O-doping is inevitable in most carbon materials due to its widespread and unavoidable presence in most precursors, its role is generally ignored and the effect of oxygen co-doping is not widely discussed.^17^ Hence, the aim of the present work was to understand the effect of O-doping along with N-doping, especially in terms of the fast-charging performance of batteries. Furthermore, anodes such as lithium titanate (LTO) and TiO_2_ have attracted significant attention for fast-charging applications. However, there are notable concerns about their limitations; specifically, the challenge of low energy density, which has been thoroughly emphasized. Despite the implementation of various innovative approaches to enhance the energy density of TiO_2_, such as incorporating crystalline Ti_2_O_1.3_(PO_4_)_1.6_ as a hybrid material with TiO_2_ (ref. 18) and carbon or utilizing 2D Ti_2_(PO_4_)_2_F nanosheets,^19^ their lower operating voltage and substantial cost still remain as significant concerns.^20^ On the other hand, graphite, derived from petroleum, has long been considered an ecologically friendly material. However, concerns about its sustainability raise questions about its viability for the future. With these concerns, hard carbon materials derived from various biomaterials, such as sucrose,^21^ glucose, cellulose,^22^ lignocellulose,^23^ are being actively investigated by researchers. However, achieving precise control over the specific proportions of the heteroatoms in such compounds is challenging due to their bio-derived nature. Also, these compounds being non-aromatic causes low char yields. Hence, in the present work we utilized a bio-based polymer, namely poly(benzimidazole-*co*-amide), as the single source of carbon, nitrogen, and oxygen for obtaining N, O co-doped hard carbons. Further, in order to understand the optimum nitrogen- and oxygen-doping balance in carbon materials suitable for fast charging, we synthesized copolymers with varying proportions of polybenzimidazole : polyamide (PBIPA) of 8.5 : 1.5, 7 : 3, and 5 : 5 and pyrolyzed them at 800 °C to obtain N, O co-doped HCs. Electrochemical studies revealed that the pyrolyzed polybenzimidazole-*co*-amide 8.5–1.5 exhibited a maximum delithiation capacity of 135 mA h g^−1^ at a current density of 4.0 A g^−1^. This underscores its suitability as the most favourable material for applications requiring fast charging, surpassing its counterparts. The detailed synthetic procedure, electrochemical studies, and discussions pertaining to it are presented in the following sections.

## Experimental section

### Materials

1.0 M LiPF_6_ in EC/DEC = 50/50 (v/v), battery grade, was purchased from Sigma-Aldrich. Battery-grade acetylene black was purchased from Denka Japan Private Co. Ltd for usage as a conductive material in the anode. Poly(vinylidene) fluoride was purchased from Sigma Aldrich. *N*-Methyl 2-pyrrolidone (NMP) was purchased from FUJIFILM Wako Pure Chemical Corporation (Wako, Japan).

### Synthesis

The detailed synthetic procedure for the polymers was reported by Kaneko *et al.*^24^ Briefly, the polymers were synthesized using 3,4-diaminobenzoic acid (3,4-DABA) and 4-aminobenzoic acid as precursors (Scheme S1†). The polymers were synthesized by maintaining precursor ratios of 9 : 1, 8.5 : 1.5, 7 : 3, 6 : 4, and 5 : 5 for obtaining copolymers PBIPA in the ratios 9 : 1, 8.5 : 1.5, 7 : 3, 6 : 4, and 5 : 5, respectively. The ratios of polymers were chosen such that there was a difference of ∼10% amide content in each polymer with the aim of obtaining varying oxygen doping contents. Wherein, in the case of PBIPA10–0 (PBI),^16^ the homopolymer was synthesized using only 3,4-DABA as the monomer, while the homopolymer polyamide 0–10 was synthesized using only 4-ABA.^25^ For both the homopolymer and copolymer, the same synthetic route was followed, *i.e.*, initially the solvent (PPA) was heated at 100 °C to remove any moisture, followed by the addition of the precursors and copolymerization at 200 °C for 14 h in a nitrogen atmosphere. The obtained polymers were then cooled to room temperature and stirred in a beaker containing distilled water to wash off any unreacted precursors. The polymers were then crushed and dispersed in 10% KOH aq. solution and the mixture was continuously stirred at room temperature overnight before filtering and washing with water until neutral pH. The resulting polymer powders were dried under vacuum at 80 °C for 8 h. The polymers were then pyrolyzed at 800 °C to obtain the carbon materials, which were named as PYPBIPA9–1, 8.5–1.5, 7–3, 6–4, 5–5, respectively. The pyrolysis was conducted in two different steps. Initially the temperature was raised to 750 °C at 5 °C min^−1^, which took 2 h 20 min, and later it was increased again to 800 °C at 1 °C min^−1^, which took 50 min. Further, the temperature was maintained at 800 °C for 1 h; hence, the total time for pyrolysis was 4 h 10 min. The pyrolyzed materials were ultrasonicated in 10% HCl solution to remove any amorphous carbon or carbonate impurities in the samples. The ultrasonication was followed by drying at 80 °C under vacuum for about 12 h to remove water.

X-Ray photoelectron spectroscopy (XPS) measurements were conducted on a Fisons instruments S-probe TM 2803 instrument. High-resolution transmission electron microscopy (HR-TEM) images were acquired using a scanning transmission electron microscope (JEM-ARM200F, JEOL Co. Ltd) at an acceleration voltage of 200 kV. Powder X-ray diffraction (XRD) studies were conducted on a Smart Lab X-ray diffractometer (Rigaku) with Cu Kα radiation (*λ* = 0.154 nm, over the 2*θ* range of 2–45° with a step size of 0.02°).

### Electrode preparation and cell fabrication

The respective carbon materials, PVDF, and acetylene black were mixed in an 8 : 1 : 1 ratio and rotated in a Kakuhunter ball mill for preparing a uniform slurry. The obtained slurry was coated on copper foils using a doctor blade while maintaining a coating thickness of 0.1 mm. The electrodes were then kept in a vacuum oven at 80 °C for about 12 h. The dried electrodes were calendared to 0.06 mm thickness at 80 °C. Electrodes of 17 mm diameter were punched out of the calendared sheets. Before performing the full-cell studies, high-loading anodic half-cell studies were performed. The high-loading anodic half-cells with ≈6.8 mg cm^−2^ loading were initially precycled for 2 cycles at 0.25 A g^−1^ before the rate studies were commenced. After that, the high-loading anodic half-cells were decrimped, and reassembled into a full cell. Similarly, for full-cell fabrication, LiNCAO as the cathode was precycled at 0.25 A g^−1^ followed by reassembling into the full cell. CR2025-type coin cells were fabricated inside an argon-filled glove box (O_2_, H_2_O <0.5 ppm) using the PYPBIPA8.5–1.5, 7–3, 5–5 based anodes, lithium foil as the counter and reference electrodes, a polypropylene separator (25 μm, Celgard), and 1.0 M LiPF_6_ (50 : 50) ethylene carbonate : diethyl carbonate (EC : DEC) as the electrolyte. For the fabrication of symmetric cells, anodic half-cells were prepared and cycled at 50 mA g^−1^ for three cycles and the cells were decrimped in an Ar-filled glovebox while the anode was in a lithiated state. The lithiated electrode and an electrode with the same composition in the unlithiated state were used to prepare symmetric cells. The symmetric cells were rested for 12 h before commencing the charge–discharge studies. After 12 h, the symmetric cells were cycled at 50 mA g^−1^ current density.

### Electrochemical measurements

All the electrochemical measurements were performed in the potential range of 0.01 V to 2.1 V (*vs.* Li/Li^+^) at 25 °C. Cyclic voltammetry was performed in a biologic VSP workstation at 0.1, 0.2, 0.4, 0.6, 0.8, and 1.0 mV s^−1^ scan rates. Potentiostatic electrochemical impedance spectroscopy (PEIS) was performed in the frequency range of 10 MHz to 0.1 Hz. Galvanostatic charge–discharge (GCD) measurements were conducted in a biologic battery cycling system at various current densities.

## Results and discussions

**Fig. 1:**
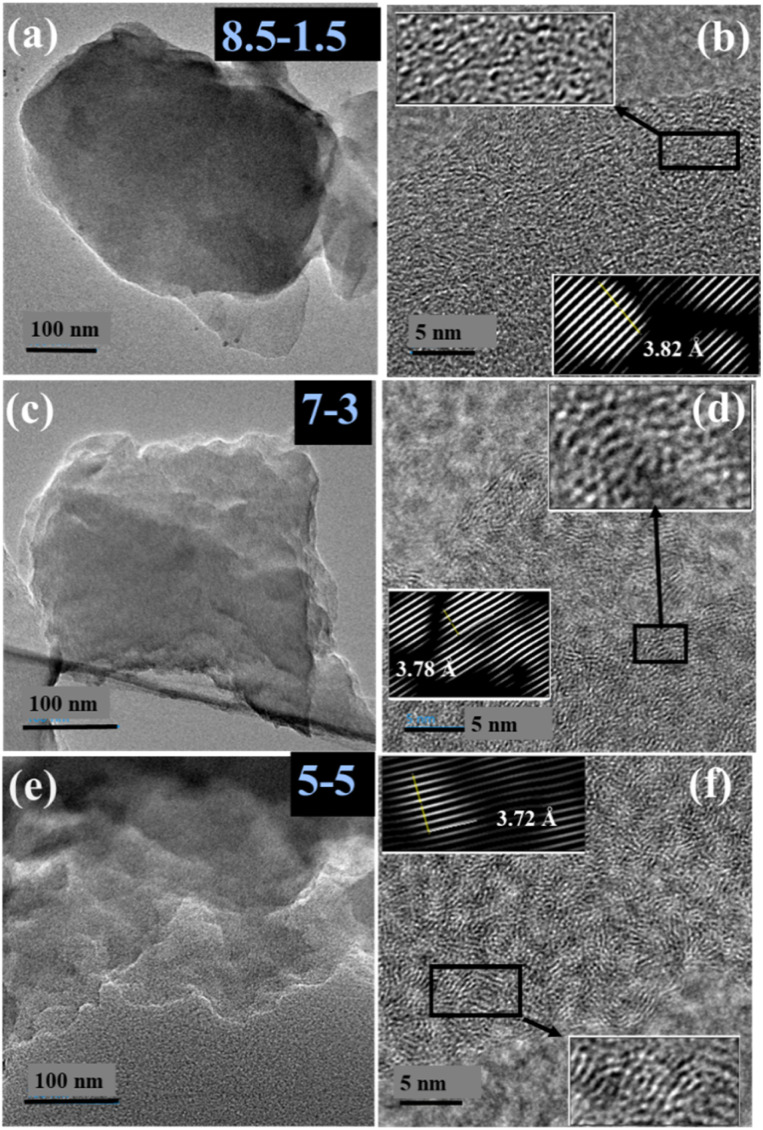
HR-TEM images of (a) 100 nm and (b) 5 nm of PYPBIPA8.5–1.5, (c) 100 nm and (d) 5 nm of PYPBIPA7–3 and (e) 100 nm and (f) 5 nm of PYPBIPA5–5.

**Fig. 2:**
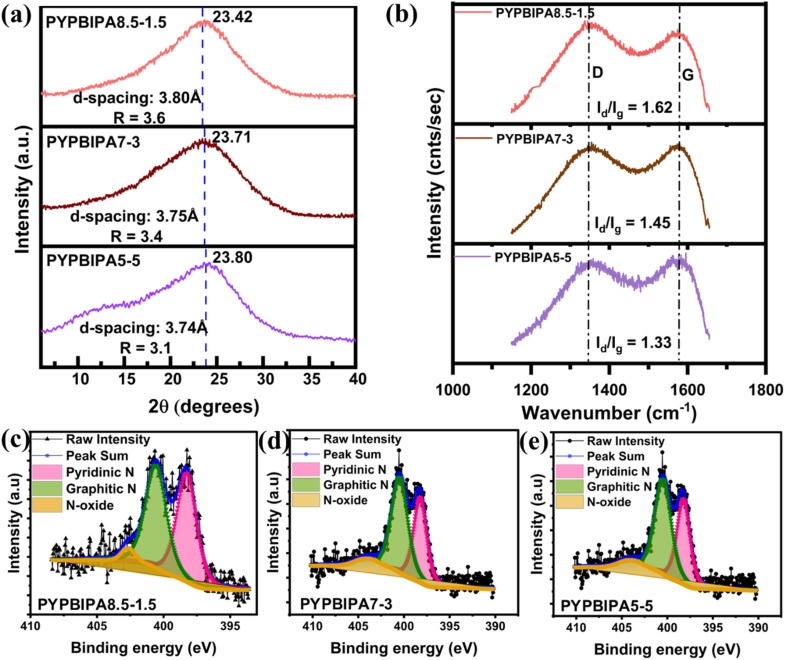
(a) Comparison of the XRD and (b) Raman spectra of PYPBIPA8.5–1.5, 7–3, and 5–5. Deconvoluted N 1s spectra of (c) PYPBIPA8.5–1.5, (d) PYPBIPA7–3, and (e) PYPBIPA5–5.

**Fig. 3:**
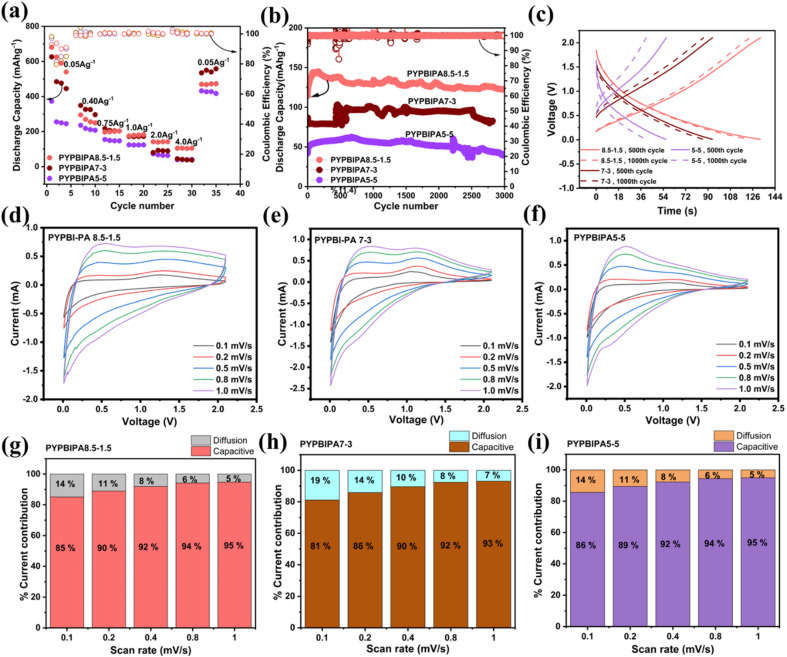
(a) Comparison of the rate and (b) comparison of the long-cycling study results for 3000 cycles. (c) GCD curves for the 500th and 1000th cycles. (d–f) CV scan rate studies and (g–i) percentage contribution of diffusion-based and capacitive-based charge-storage currents for PYPBIPA8.5–1.5, 7–3 and 5–5, respectively.

The morphological analysis of the PBIPA-derived carbon materials was initially performed using HR-TEM. The HR-TEM images at a low magnification of 100 nm indicated a layered morphology for all three samples, as shown in Fig. 1a, c, and e. The high-magnification HR-TEM images at 5 nm (zoomed-in images in the insets of Fig. 1b, d and f) indicated a disordered arrangement of carbon planes for all three samples. To shed more light on the spacing between the carbon planes, the associated HR-TEM images were subjected to Fourier-transform (FT) and inverse FT (IFT) studies for determining the interplanar spacings, which were found to be 3.82, 3.78, and 3.72 Å for PYPBIPA8.5–1.5, 7–3, and 5–5, respectively, as shown in Fig. 1, which were in close agreement with the *d*-spacing determined using XRD. Further, TEM-EDX mapping of all three samples indicated the uniform distribution of carbon, nitrogen, and oxygen, as indicated in Fig. S2–S4† for PYPBIPA8.5–1.5, 7–3, and 5–5, respectively. The XRD plots for PYPBIPA8.5–1.5, 7–3, and 5–5 shown in Fig. 2a–c, respectively, indicated broad peaks at 23.42°, 23.71°, 23.80° corresponding to 002 reflections. The *d*-spacing of the samples was calculated using Bragg's law,^26^ which were found to be 3.80, 3.75, and 3.74 Å for PYPBIPA8.5–1.5, 7–3, and 5–5 in comparison to 3.6 Å for PYPBI800 as reported in our previous research work.^16^ PYPBI800 will be henceforth referred to as PYPBIPA10–0, as it was obtained by pyrolyzing pure polybenzimidazole without any polyamide group. Hence, these results clearly indicated that the inclusion of oxygen alongside nitrogen led to an increased *d*-spacing due to the larger size of O atoms.^16^ Further, the *R* factor was also deduced using the XRD plots, which is an indicator of the relative number of carbon sheets arranged in parallel in a given crystal. The *R* factor was calculated by dividing the ratio of the background to the ratio of the signal^27^ (Fig. S1†), which was found to be 3.2 in the case of 10–0 material and followed the order PYPBIPA8.5–1.5 > 7–3 > 5–5 > 10–0 (Fig. 2a); thus indicating the relatively greater stacking of carbon sheets in PYPBI8.5–1.5 than in the others. This can be explained based on the H-bonding strength between the adjacent chains of the polymers used for the synthesis of the hard carbons. As reported by Kaneko *et al.*,^24^ the incorporation of small amounts of amide groups to the polybenzimidazole backbone enhances the interchain H-bonding strength between adjacent chains. This signified the relatively weaker H-bonding in the case of the pure polybenzimidazole than the copolymers. Also, according to their report, amide incorporation beyond a particular limit, *i.e.*, 20%, weakened the interchain H-bonding due to the presence of excessive amide groups, which led to too many amide–imidazole and amide–amide interactions. This would summarize the H-bonding strength of the copolymers to be in the order PBIPA8.5–1.5 > 7–3 > 5–5. Thereafter upon pyrolysis, polymers with a stronger H-bonding resulted in a higher stacking of carbon planes, which further led to greater *R* factors. This explains the possible reason for the similar trend of both the *R* factor of the carbon materials and H-bonding strength of the polymers. The higher stacking of carbon planes in the case of PYPBIPA8.5–1.5 could be another advantage for the less hindered movement of Li ions within the carbon material. To gain a greater understanding of the defects present in both materials, Raman spectroscopy was conducted (Fig. 2b). The Raman spectra of all three samples exhibited two conventional peaks at similar Raman shifts of ∼1350 and ∼1570 cm^−1^, which could be identified as the D band (defect-induced mode) and G band (E_2g_ mode), respectively.^28^ The ratio of the D band to G band, *i.e.*, the *I*_d_/*I*_g_ ratio, was found by dividing the integrated area under the D peak to integrated area under the G peak.^29^ The *I*_d_/*I*_g_ ratios were found to be 1.62, 1.45, and 1.33 for PYPBIPA8.5–1.5, 7–3, and 5–5, respectively; hence indicating the large number of defect sites in all three materials. The higher number of defect sites can be another advantage for PYPBI8.5–1.5 during fast charge–discharge as Li ions can easily move from one plane to another plane in the perpendicular direction. Also, these defect sites can act as active sites for Li-ion adsorption. Following morphological analysis, elemental analysis was conducted using XPS, which indicated the presence of carbon, nitrogen, and oxygen in all three samples (Fig. S5†). The contents of nitrogen were determined to be 11.2, 9.9, 8.0 at% while the oxygen contents were found to be 13.1, 20.0, and 25.0 at% in PYPBIPA8.5–1.5, 7–3, and 5–5, respectively (Table S1†). The nitrogen and oxygen contents in 10–0 were reported to be 14.8 at%^16^ and 0.6 at%, respectively. These findings illustrate that the nitrogen content diminished as the proportion of the polybenzimidazole group decreased, while the oxygen content rose with the increasing proportion of the polyamide group. Also, the poly(benzimidazole-*co*-amide) copolymers acted as a single source of carbon and nitrogen, as well as oxygen. The benzimidazole unit contains a benzene ring and an imidazole ring, wherein the imidazole ring with covalently bonded nitrogen atoms can contribute toward nitrogen doping. The presence of these nitrogen-containing aromatic units confers the polybenzimidazole backbone with high thermal resistance, thereby leading to high nitrogen doping in the obtained carbon materials when pyrolyzed under an inert atmosphere.^30^ Whereas polyamide with an amide (NH–CO) functional group in its backbone can decompose more easily and most of the nitrogen is lost during carbonization, resulting in oxygen doping in the remaining carbon material. To substantiate the contributions of the benzimidazole and amide units to the resultant carbon material, a homopolymer polyamide (PA) was synthesized and consequently subjected to pyrolysis, and is henceforth referred to as PYPBIPA0–10. XPS analysis of PYPBIPA0–10 (Fig. S5 and Table S1†) indicated the content of nitrogen to be merely 2.6 at% while the content of oxygen was as high as 31.3 at%. These results clearly indicated that the benzimidazole unit primarily contributed to nitrogen doping, while the amide unit played a major role in oxygen doping. The benefits of nitrogen doping on the fast-charging performance of LIBs were already discussed in our previous research work.^16^ This study was undertaken with the primary objective of elucidating the impact of oxygen doping in conjunction with nitrogen doping, particularly with respect to the fast-charging performance of batteries. Compared to single heteroatom doping, co-doping makes a significant contribution to the electrical conductivity, interlayer spacing, and ion-storage capacity due to the synergistic effects of both elements; whereby N-doping can induce defect sites, introduce active sites for surface redox reactions, and facilitate electronic conductivity^31^ while O-doping creates oxygen-based functional groups, such as –C=O, which can reversibly convert to –C–O upon lithiation, thus increasing the gravimetric capacity through surface redox reactions.^32^ Also, N- and O-doping can induce defect sites, which can bind strongly with inserted Li ions. Further they can be electron donors to the carbon planes, thus inducing strong bonding between the Li ions and the graphene sheets. Indeed, both nitrogen (N) doping and combined nitrogen and oxygen (N,O)-doping contribute to the enlargement of the *d*-spacing in carbon materials. This expanded *d*-spacing can facilitate the swift movement of Li-ions for insertion between the layers. To gain a detailed understanding of the functional groups present in the carbon materials, the N 1s, O 1s, and C 1s spectra were deconvoluted for all three samples. Upon deconvolution, nitrogen was detected in three different forms, *i.e.*, pyridinic nitrogen, graphitic nitrogen, and N-oxide at 398 ± 0.3, 400 ± 0.3, and 402 ± 0.3 eV,^33^ similar to 10–0 except for a minute content of pyrrolic nitrogen (0.50 at%).^16^ The deconvolution of the O 1s spectra indicated the presence of C–O/C=O and absorbed oxygen at 530 ± 0.3 and 532 ± 0.3 eV, respectively, for all the samples, as shown in Fig. S8.† The polarity of the C=O bond significantly enhances the Li-ion-storage capacity by facilitating reversible redox reactions during lithiation and delithiation cycles. The redox process involving the carbonyl group and lithium ions can be summarized as –C=O + Li^+^ + e^−^ = –C–O–Li. Previous studies have investigated the interaction of C=O with Li ions. One such study by Sun *et al.*^34^ demonstrated this using density functional theory (DFT) to study the lithiation and delithiation cycles in a modified covalent organic framework (COF) with azo and carbonyl groups. Their analysis, based on the energy differences between the pristine and lithiated COF structures, revealed a preferential interaction of Li^+^ ions with the C=O groups. Additionally, they used *in situ* Fourier-transform infrared (FT-IR) spectroscopy, which indicated an increase in C=O peak intensity with increasing the state of discharge, suggesting Li–O bond formation. Furthermore, XPS measurements also displayed a new peak corresponding to Li–O bond formation in addition to the existing C=O peaks. While many studies have shown the effectiveness of oxygen-based functional groups in lithium-storage applications, the high steric hindrance and reduced conductivity due to excessive O-doping can be a major drawback. Hence this research work mainly focused on determining the optimal amount of oxygen in the anodic active materials suitable for the fast charging of LIBs. Following the morphological and elemental analysis, electrochemical studies were conducted using CR2025-type coin cells fabricated under an Ar atmosphere (O_2_ and H_2_O <0.5 ppm). Initially, the batteries were subjected to cyclic voltammetry at a scan rate of 0.1 mV s^−1^, as shown in Fig. S10a–c.† The cyclic voltammograms of all three anodic half-cells indicated broad peaks, which could be due to lithiation and delithiation of the disorderedly arranged carbon planes at a wide range of potentials. Further, potentiostatic electrochemical impedance spectroscopy (PEIS) was conducted before and after the cyclic voltammetry tests in order to compare the resistance.^35^ After cyclic voltammetry, the resistance decreased drastically from 107 Ohms to 45 Ohms, 122 Ohms to 24 Ohms, and 96 Ohms to 22 Ohms in the cases of PYPBIPA8.5–1.5, 7–3, and 5–5, respectively, as indicated in Fig. S11.† The decrease in resistance observed after the cyclic voltammetry (CV) could be ascribed to the development of a solid electrolyte interphase (SEI) at the electrode/electrolyte interface during cycling. This SEI formation facilitated more efficient charge transfer and enhanced the electrical conductivity.^36^ The SEI formation consumed active lithium, leading to an overall loss of lithium. The first cycle discharge(lithiation)/charge(delithiation) capacities also clearly indicated SEI formation.^37,38^ When cycled at a current density of 0.25 A g^−1^ as shown in Fig. S12,† initial coulombic efficiencies (ICE) of 61%, 60%, and 51% were obtained for PYPBIPA8.5–1.5, 7–3, and 5–5, respectively, which were comparatively lower than the 68% ICE of 10–0.^16^ The lower ICE in the case of the PYPBIPA-derived carbons could be due to the presence of C–O bonds, which react with Li ions irreversibly;^39^ however, the difference was quite low, *i.e.*, only 8%. Thereafter, galvanostatic charge–discharge studies were performed for all three samples to obtain a detailed understanding of their electrochemical performance. All the charge–discharge studies were conducted by maintaining the same current densities during both lithiation and delithiation. As shown in Fig. 3a, initially rate studies were performed and delithiation capacities of 600, 256, 199, 180, 140, and 100 mA h g^−1^ were observed at 0.05, 0.40, 0.75, 1.00, 2.00, and 4.00 A g^−1^ current densities for PYPBIPA8.5–1.5. Further, in the case of PYPBIPA7–3, delithiation capacities of 500, 325, 204, 171, 90, and 38 mA h g^−1^ were obtained at 0.05, 0.40, 0.75, 1.00, 2.00, and 4.00 A g^−1^, respectively, and in the case of PYPBIPA5–5, delithiation capacities of 211, 147, 123, 64, and 39 mA h g^−1^ were obtained at 0.05, 0.40, 0.75, 1.00, 2.00, and 4.00 A g^−1^ current densities. At lower current densities of 0.40 and 0.75 A g^−1^, both PYPBIPA7–3 and PYPBIPA8.5–1.5 showed similar discharge capacities; however, at higher current densities of 2.0 and 4.0 A g^−1^, PYPBIPA8.5–1.5 showed better performance. Furthermore, PYPBIPA8.5–1.5 outmatched 10–0,^16^ which showed discharge capacities of 206, 168, and 125 mA h g^−1^ at 0.37, 0.74, and 1.86 A g^−1^, respectively. Subsequently, to understand whether PYPBIPA8.5–1.5 can retain its high delithiation capability even during long cycling, charge–discharge studies were conducted at 4.00 A g^−1^ for 3000 cycles. Even during long cycling, PYPBIPA8.5–1.5 showed a much higher delithiation capacity of 135 mA h g^−1^ compared to 86, 100, and 60 mA h g^−1^ for PYPBIPA10–0, 7–3, and 5–5, respectively. The GCD plots during cycling are shown in Fig. 3c. The charge–discharge plots of these materials are shown in Fig. 3c and clearly indicate a sloping region throughout the voltage window. Therefore, an adsorption-type Li-ion-storage mechanism can be proposed for these materials, which is very common in the case of heteroatom-doped hard carbons^40^ Even in terms of capacity retention, PYPBIPA8.5–1.5 surpassed the others. The capacity retention of PYPBIPA8.5–1.5 after 1000 cycles was found to be 95%, 93% after 2000 cycles, and 90% after 3000 cycles, which is commendable, whereas the capacity retention in the case of 7–3 was 83.3%, and for 5–5 it was 65.5% after 3000 cycles. PYPBIPA8.5–1.5 with an optimum balance of nitrogen and oxygen content outperformed all the others during the rate studies as well as long-cycling studies. From the long-cycling studies, in terms of rate capability, reversible capacity, and capacity retention, the following trend was observed 8.5–1.5 > 7–3 > 5–5. To further validate this decreasing trend of parameters with increasing polyamide composition, carbon materials from intermediate polyamide compositions, such as 9–1 (intermediate to 100–0 and 8.5–1.5) and 6–4 (intermediate to 7–3 and 5–5) were synthesized and applied as anodic active materials in LIBs. The XPS analysis results of these carbon materials are summarized in Table S1.† The deconvoluted N 1s, O 1s, and C 1s spectra of PYPBIPA9–1 and PYPBIPA6–4 are presented in Fig. S7–S9,† respectively. Comparisons of the rate studies and long-cycling results of these anode materials are shown in Fig. S14 and S15,† respectively. During the rate studies, PYPBIPA9–1 exhibited discharge capacities of 316, 235, 167, 130, 111, and 94 mA h g^−1^ at current densities of 0.05, 0.40, 0.75, 1.00, 2.00, and 4.00 A g^−1^, respectively. PYPBIPA6–4 showed discharge capacities of 262, 222, 140, 130, 80, and 48 mA h g^−1^ at current densities of 0.05, 0.40, 0.75, 1.00, 2.00, and 4.00 A g^−1^. During the long-cycling studies at 4.0 A g^−1^, PYPBIPA9–1 delivered a discharge capacity of 122 mA h g^−1^ with a capacity retention of 90%, whereas PYPBIPA6–4 delivered 74 mA h g^−1^ with a capacity retention of 76%. Furthermore, the difference in discharge capacities between PYPBIPA9–1 and PYPBIPA8.5–1.5 was observed to be minimal, *i.e.*, 13 mA h g^−1^, attributed to their similar nitrogen and oxygen contents. These results indicated that the trend in rate capability, reversible capacity, and capacity retention was consistent even with the inclusion of 9–1 and 6–4 among the 8.5–1.5, 7–3, and 5–5 compositions. These results underscore the critical role of optimizing the nitrogen- and oxygen-doping levels, as shown by PYPBIPA9–1 and PYPBIPA8.5–1.5, to achieve high performance, especially under high current rate conditions. Next, symmetric cells were fabricated using PYPBIPA5–5, PYPBIPA6–4, PYPBIPA7–3, PYPBIPA8.5–1.5, and PYPBIPA9–1 active materials. Electrochemical studies using the symmetric cells enabled a greater understanding of the parasitic reactions that could lead to capacity loss during cycling. In a typical anodic half-cell, lithium loss due to parasitic reactions does not necessarily translate into a loss of capacity as the lithium inventory is high.^41^ Further in full cells, due to the applied potential window, oxidative side reactions on the cathodic side affect the capacity retention. Hence in both cases of anodic half-cells and full-cells, a particular understanding of the anode is difficult. In this regard, symmetric cells enable a deeper understanding of the anode (or electrode of interest) as the lithium inventory is limited and there is very limited scope for parasitic reactions involving the electrolyte in the potential window of ±0.5 V.^41^ Symmetric cell charge–discharge studies of PYPBIPA5–5, 7–3, 6–4, 8.5–1.5, and 9–1 were conducted and the results thereof indicated that PYPAPBI8.5–1.5 exhibited stable cycling behaviour (Fig. S16 and S17d†) with the highest capacity retention of 91.3% after 25 cycles, among the others. The trend of capacity retention for all the samples is summarized in Table S4.† This is consistent with the stable cycling behaviour observed in the long-cycling studies of the anodic half-cell. Hence, these results clearly indicate that PYPBIPA8.5–1.5 had the best performance among the others. Furthermore, to gain a detailed understanding of the effect of oxygen on the charge–discharge performance, pyrolyzed polyamide homopolymer (henceforth referred to as PYPBIPA0–10)-based anodes were also studied. The XPS analysis and charge–discharge performance (rate studies and long-cycling studies) of PYPBIPA0–10 are shown in Fig. S5 and S13,† respectively. The XPS analysis of PYPBIPA0–10 indicated the content of N was 2.6 at% and the O content was 31.3 at% in comparison to 14.6 at% with nitrogen doping and 0.6 at% with oxygen doping in PYPBIPA10–0. The impact of the N and O contents was directly reflected in the performance of the battery. As we observed in the rate studies (Fig. S13†), PYPBIPA0–10 delivered discharge capacities of 152, 125, and 85 mA h g^−1^ in contrast to 206, 168, and 125 mA h g^−1^ for PYPBIPA10–0 at 0.4, 0.75, and 2.0 A g^−1^ (Table S2†). Furthermore, long cycling at 4.0 A g^−1^ showed a discharge capacity of 51 mA h/g in the case of PYPBIPA0–10, whereas it was reported to be 86 mA h g^−1^ in the case of PYPBIPA10–0.^16^ These results clearly indicated that PYPBIPA10–0 with the maximum nitrogen doping demonstrated a better performance than PYPBIPA0–10 with maximum oxygen doping in terms of its discharge capacity as well as its long-cycling ability.^45^ Compared to PYPBIPA8.5–1.5, 7–3, and 5–5, PYPBIPA0–10 exhibited an inferior discharge capacity and unsatisfactory cycle performance (Fig. S13 and Table S3†). The reason could be excessive oxygen doping. In general, an increase in oxygen content leads to disadvantages, as it can result in irreversible capacity loss, leading to poor cycle performance, specifically low-capacity retention. This phenomenon is evident in the cases of PYPBIPA5–5 and 0–10. As mentioned earlier, PYPBIPA8.5–1.5 outperformed 10–0 due to the co-doping effect of oxygen alongside nitrogen. However, this favourable effect was not observed in the cases of PYPBIPA7–3, 5–5, and 0–10. It was noteworthy that PYPBIPA0–10, despite having a high level of oxygen doping, was unable to rival the performance of PYPBIPA10–0 and other carbon materials. While oxygen co-doping can offer advantages, this study noted that an increase in polyamide content beyond 15% for obtaining N, O co-doped carbons had a detrimental effect on battery performance. This is attributed to steric hindrance caused by the bulky oxygen groups, impeding the diffusion of lithium ions, consequently resulting in a diminished electrochemical performance. These results clearly indicated that PYPBIPA8.5–1.5 was the best-performing material among the others, demonstrating both rapid charge–discharge capabilities and a long-cycling capability. In this context, it is crucial to highlight that while oxygen (O) co-doping contributes significantly to the fast-charging performance of batteries, achieving an optimal balance between both elements is essential. Further, to understand the mechanism of charge storage, kinetic studies were conducted using cyclic voltammetry at scan rates of 0.1, 0.3, 0.5, 0.7, and 1.0 mV s^−1^ for all three samples. The cyclic voltammograms of all three samples are shown in Fig. 3c, d, and e. The Power law below^42^

1 *i* = *av*^*b*^

was utilized, where *i* signifies the current, and *v* signifies the scan rate. The value of *a* and *b* are obtained from the intercept and slope of a log *i vs.* log ν plot, respectively. The linear fit of the logarithmic plots of all three samples are shown in Fig. S18.† The value of *b* determines whether a diffusion-controlled charge-storage mechanism is dominant, or a capacitive-controlled charge-storage mechanism is dominant. When *b* = 0.5, a diffusion-controlled mechanism is known to happen, whereas when *b* = 1.0, a surface capacitive-controlled charge-storage mechanism is known to happen. In the case of PYPBIPA8.5–1.5, 7–3 and 5–5, the values of *b* were found to be 0.98, 0.97, and 0.97, respectively, which were all higher than the *b* value of 0.90 (ref. 16) for 10–0. These results suggest that a substantial capacitive-controlled charge-storage mechanism predominated over a diffusion-controlled mechanism for all the carbon samples. The value of *b* was constantly higher in all the N and O co-doped carbons than in the purely N-doped carbon, which was likely due to the availability of a large number of surface redox sites in N, O co-doped carbons. Furthermore, in order to quantitatively distinguish the capacitive and diffusion charge-storage contributions, eqn (2), *i.e.*,^43^

**Fig. 4:**
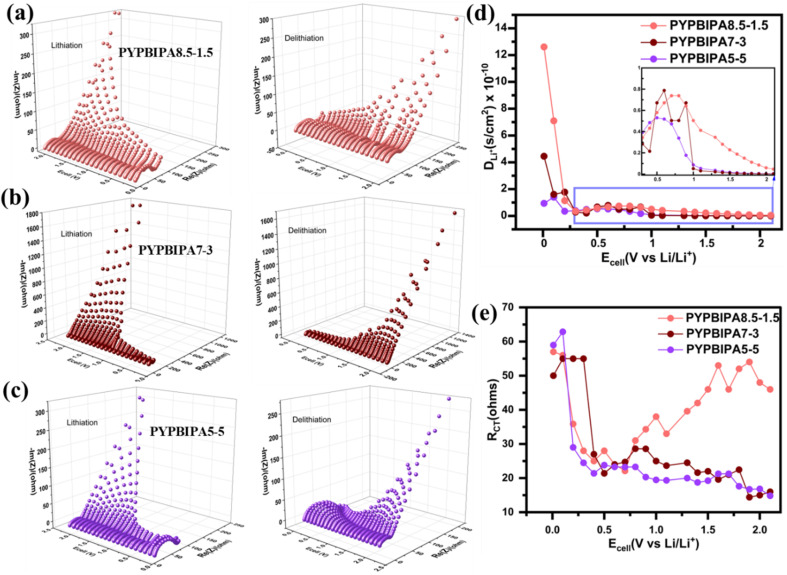
DEIS plots for lithiation (left) and delithiation (right) for PYPBIPA (a) 8.5–1.5, (b) 7–3, and (c) 5–5 (d)comparison of lithium-ion diffusion coefficient and (e)comparison of charge transfer resistance for PYPBIPA8.5–1.5, 7–3 and 5–5 in the voltage range of 2.1 to 0.01 V during lithiation.

2 *i* = *k*_1_*v* + *k*_2_*v*^1/2^

was used, where the redox current *i* is a cumulative measure of the capacitive-based charge storage *k*_1_*ν* and the diffusion-based charge storage *k*_2_*ν*^1/2^ at each scan rate. The *k*_1_ and *k*_2_ values were determined using a linear fit of *i*/*v*^1/2^*vs. v*^1/2^ as shown in Fig. S19† for all three carbon materials. Upon calculation of the capacitive-based current and diffusion-based current contributions, it was found that all three samples showed major contributions to the capacitive-based currents at all scan rates. The percentage contributions of the capacitive current were found to be 85%, 90%, 92%, 94%, and 95% for PYPBIPA8.5–1.5, whereas they were found to be 81%, 86%, 90%, 92%, and 93% in the case of 7–3, and 86%, 89%, 92%, 94%, and 95% in case of 5–5 at 0.1, 0.2, 0.4, 0.8, and 1.0 mV s^−1^, respectively, as shown in Fig. 3f–h. The capacitive current contributions were reported to be 78%, 86%, 89%, 91%, and 92% at 0.1, 0.2, 0.4, 0.8, and 1.0 mV s^−1^, respectively, for 10–0.^16^ The above results clearly demonstrated that the capacitive current contribution was constantly higher in the case of the co-doped carbons than that of the purely nitrogen-doped carbon at all scan rates, thus indicating that the inclusion of oxygen along with nitrogen led to an increased number of surface redox sites. Though the O content was observed to be higher in PYPBIPA7–3 and 5–5, implying the presence of a greater number of active sites for surface redox reactions, they did not show higher delithiation capacities than PYPBIPA8.5–1.5 since the bulky O groups would have reduced Li-ion diffusion within the carbon planes. In order to validate this, the lithium-ion diffusion coefficient was calculated using dynamic electrochemical impedance spectroscopy (DEIS) in the potential range of 0.01 V to 2.1 V for PYPBIPA8.5–1.5, 7–3, and 5–5, as shown in Fig. 4a–c. The DEIS profiles of all three carbon materials showed typical behaviours, *i.e.*, a semicircle in the high-frequency region followed by a straight line in the low-frequency region.^44^ The lithium-ion diffusion coefficient (*D*_Li^+^_) was evaluated with the impedance data obtained at lower frequencies using the following Warburg equation:^45^3
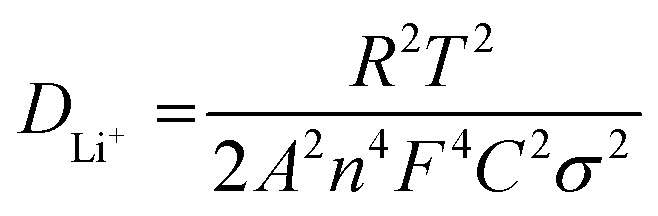
where *R* is a universal gas constant, *T* is the temperature in Kelvin, *A* is area of the electrode (cm^2^), *n* is the number of electrons transferred, *F* is Faradays constant, *C* is the concentration of the electrolyte, and *σ* is Warburg's coefficient. The Warburg coefficient is evaluated using the following equation:^46^

**Fig. 5:**
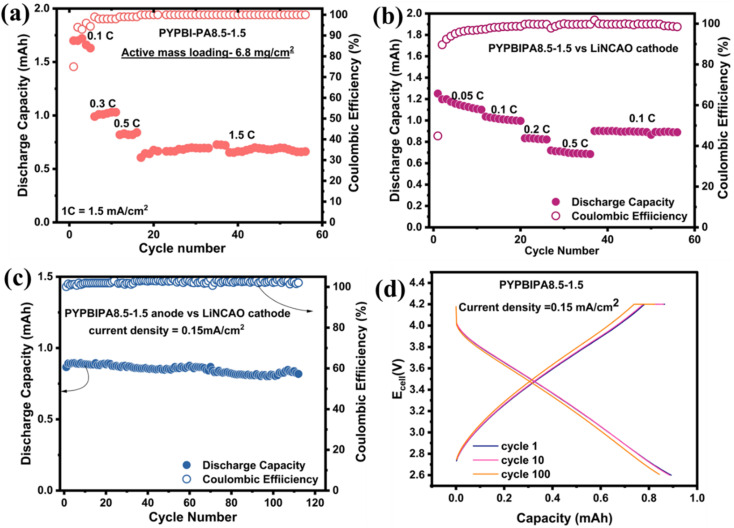
(a) Rate studies and long cycling of the PYPBIPA8.5–1.5 based anodic half-cell at a high loading of 6.8 mg cm^−2^: (b) rate studies and (c) long cycling. (d) Charge discharge plots of PYPBIPA8.5–1.5 *vs.* the LiNCAO cathode full cell at 0.15 mA cm^−2^.

4 *Z*_real_ = (*R*_electrolyte_ + *R*_CT_) + *σω*^−0.5^

where *Z*_real_ is the real impedance and *ω* is the angular frequency. The angular frequency *ω* was calculated as 2π*f*, where *f* corresponds to the values of frequency in the low-frequency region. Fig. 4d provides a comprehensive comparison of the lithium-ion diffusion coefficient for all three samples during lithiation at each potential within the range of 0.01 to 2.1 V. The lithium-ion diffusion coefficient was observed to be highest in the case of PYPBIPA8.5–1.5, following the order PYPBIPA8.5–1.5 > 7–3 > 5–5. As previously discussed, this data corroborated the impact of excessive oxygen doping, wherein the presence of bulky oxygen groups increased the steric hindrance and consequently, ion diffusion was diminished in PYPBIPA7–3 and 5–5. At faster current rates, which is marked by a significant influx of Li ions, the diminished lithium-ion diffusion translated into a lower Li-ion-storage capability in PYPBIPA7–3 and 5–5. Furthermore, the comparison also clarified that there was no significant difference in diffusion coefficients for PYPBIPA7–3 and 5–5 in between 1 and 2.1 V. However, beyond 1 V down to 0.01 V, there was an increase in the lithium-ion diffusion coefficient for PYPBIPA7–3. This suggests that, in the case of PYPBIPA7–3, lithiation became more favourable within the range of 0.01 to 1 V. In contrast, for PYPBIPA8.5–1.5, the lithium-ion diffusion coefficient remained significantly high across the entire potential range of 0.01 to 2.1 V. This resulted in a notably elevated lithium-ion-storage capability, even at a very high current density of 4.0 A g^−1^. Further, upon fitting the EIS results, the charge-transfer resistance, which is generally observed at high frequencies, was obtained for all three samples in the potential range of 0.01 to 2.1 V during lithiation. Fig. 4e showcases plots for comparison of the charge-transfer resistance for all three samples during lithiation. The charge-transfer resistance was observed to be the maximum for PYPBIPA8.5–1.5 followed by PYPBIPA7–3 and PYPBIPA5–5. This could be due to differences in the oxygen-doping content. In general, oxygen being highly polar facilitates charge transfer. Hence, PYPBIPA5–5 containing the maximum oxygen content faced the relatively least resistance, whereas PYPBIPA8.5–1.5 with the minimum oxygen content encountered relatively higher resistance, while PYPBIPA7–3 with an intermediate oxygen content faced intermediate resistance. Taking a holistic view, it is evident that while PYPBIPA8.5–1.5 exhibited a slightly higher charge-transfer resistance owing to its lower oxygen content, this was effectively compensated for by its faster lithium-ion diffusion rate. This compensation was clearly reflected in its superior fast-charging capability. Further, the coin cells cycled for 3000 cycles were decrimped inside an Ar-filled glove box. X-Rays can penetrate up to 10 nm of the material surface, hence they can easily detect the SEI components that are generally present within this limit. Thus, the cycled anodes were further subjected to XPS for understanding the various components of the SEI. The initial XPS survey spectrum detected C, O, and F in the case of all three cycled anodes. Further deconvolution was performed for understanding the various functional groups present. The deconvoluted C 1s spectrum of the cycled PYPBIPA8.5–1.5 anode (Fig. S20a†) showed peaks at 285.8, 287.5, 289.5, and 290.8 eV corresponding to C=C, C=O/C–O, Li_2_CO_3_, and C–F, respectively.^47^ The O 1s spectrum (Fig. S20b†) showed peaks at 532.7 and 533.3 eV, corresponding to C=O and C–O–C.^47^ The F 1s spectrum (Fig. S20c†) showed peaks at 685.8 and 687.9 eV, corresponding to LiF and Li_*x*_PF_*y*_.^47,48^ The XPS results suggested that Li_2_CO_3_, LiF, and Li_*x*_PF_*y*_ were the major components of the SEI in the case of the cycled PYPBIPA8.5–1.5 electrode. The C 1s spectrum of the cycled PYPBIPA7–3 anode (Fig. S21a†) displayed peaks at 285.6, 284.6, 286.9, 284.4, and 290.5 eV, corresponding to –C=C, –C=O/C–N, Li_2_CO_3_, and –C–F, respectively. The O 1s spectrum of the cycled anode (Fig. S21b†) displayed peaks at 532.2, 532.7, and 533.4 eV, corresponding to –C=O, –C–O, and –C–O–C. The F 1s spectrum of the cycled anode (Fig. S21c†) showed peaks at 687.3 and 688.17 eV, corresponding to LiF and Li_*x*_PF_*y*_, respectively. Further the deconvoluted C 1s spectrum of the cycled PYPBIPA5–5 anode showed peaks at 285.5, 286.16,286.93, and 287.75 eV, corresponding to –C=C, –C=O/C–O, Li_2_CO_3_, and C–F, respectively, as given in Fig. S22a.† The O 1s spectrum of the cycled anode (Fig. S22b†) displayed peaks at 532.2, 532.7, and 533.3 eV, corresponding to –C=O, –C–O, and C–O–C. The F 1s spectrum of the cycled anode (Fig. S22c†) showed peaks at 685.7 and 688.0 eV, corresponding to LiF and Li_*x*_PF_*y*_. Further, intrigued by the excellent performance of PYPBIPA8.5–1.5, anodic half-cells were fabricated at a high mass loading of ≈6.8 mg cm^−2^. The high-loading anodic half-cell was cycled at various current densities of 0.1 C, 0.3 C, 0.5 C, and 1.5 C, wherein 1.0 C signifies 1.5 mA cm^−2^. It showed a high initial capacity of 1.72 mA h followed by 1.0, 0.82, and 0.7 mA h at 0.1 C, 0.3 C, 0.5 C, and 1.5 C, followed by 40 cycles at 1.5C. To gain a more comprehensive understanding of the full-cell performance, coin cells were assembled utilizing anodes subjected to rate studies at high loading levels and cathodes from LiNCAO cycled for 5 cycles at 0.15 mA cm^−2^. Charge–discharge studies of the full cell were performed in the voltage window of 2.6–4.2 V in the constant current–constant voltage (CCCV) mode while maintaining constant voltage for 50 min. Initially, rate studies were conducted for the full cell, which indicated reversible capacities of 1.1, 1.0, 0.85, 0.72 mA h at 0.05 C, 0.1 C, 0.2 C, 0.5 C. Further, when the cell was cycled back at 0.1 C, a reversible capacity of 0.95 mA h could be still obtained. Further long-cycling studies of the full cell were performed, as shown in Fig. 5c. The long-cycling studies were continued immediately after the rate studies. During the long cycling, an initial reversible capacity of 0.85 mA h was obtained, which was retained up to 98% after 50 cycles and up to 95% after 100 cycles, thus indicating the high stability of the material. Fig. 5d presents the charge–discharge cycles of the full cell. These above results clearly indicated the commercial competence of PYPBI8.5–1.5. With further improved anodic loading and other parameters, a high capacity could be obtained using PYPBIPA8.5–1.5 as an anode material in the full cell.

## Conclusions

In conclusion, this study presents a novel class of fast-charging anodes for lithium-ion batteries, derived from bio-based polymers dual-doped with nitrogen and oxygen. Several distinct copolymers, namely PYPBIPA9–1, 8.5–1.5, 7–3, 6–4, and 5–5, were used to synthesize nitrogen and oxygen co-doped carbons, wherein a progressive increase in oxygen content from 13.1 at% to 20 at% and further to 25 at% was observed, corresponding to an increase in the polyamide ratio in the copolymer from 1.5 to 3.0 to 5.0. PYPBIPA8.5–1.5 demonstrated impressive long-cycling performance, delivering a delithiation capacity of 135 mA h g^−1^ over 3000 cycles with a capacity retention of 90%. Notably, it outperformed the purely nitrogen-doped carbon previously reported by our group [1]. This highlighted the clear advantage of incorporating oxygen alongside nitrogen to achieve optimal performance, especially in fast-charging applications. However, the observed variations in oxygen-doping content had a significant impact on the final battery performance. The electrochemical investigations consistently identified PYPBIPA8.5–1.5, characterized by an optimal balance of nitrogen and oxygen doping, as the superior performer among those tested. While the oxygen content was higher in PYPBIPA7–3 and 5–5, which had more surface redox sites for lithiation, they did not exhibit higher delithiation capacities compared to PYPBIPA8.5–1.5. This was due to the hindrance caused by the bulky oxygen groups, which impeded lithium-ion diffusion within the carbon planes. This phenomenon was confirmed through the evaluation of the lithium-ion-diffusion coefficient, which was found to be highest in the case of PYPBIPA8.5–1.5, following the order PYPBIPA8.5–1.5 > 7–3 > 5–5. Further analysis of the DEIS impedance data revealed that the charge-transfer resistance was highest in the PYPBIPA8.5–1.5, intermediate in 7–3, and lowest in 5–5, which could be correlated with the oxygen-doping content in the final carbon materials. Given the polar nature of oxygen, PYPBIPA5–5, with maximum oxygen doping, facilitated easier charge transfer. Taking a holistic view, it was evident that while PYPBIPA8.5–1.5 exhibited a slightly higher charge-transfer resistance owing to its lower oxygen content, this was effectively compensated for by its faster lithium-ion-diffusion rate. This was clearly reflected in its superior fast-charging capability. Hence, this study underscores the critical importance of balancing the nitrogen and oxygen contents to achieve optimal electrochemical performance, especially during charge–discharge operations at higher current densities.

## Conflicts of interest

There are no conflicts to declare.

## Supplementary Material

NA-006-D4NA00416G-s001

## Data Availability

The data supporting this article have been included as part of the ESI.† Details of synthesis have been provided in the ESI.† Additionally, the authors have included all the required information supporting the experiments referenced in the manuscript within the ESI.† The authors have also ensured to provide details related to the Results and discussions section in the ESI,† offering the readers additional figures and tables that can enhance the understanding of the scientific findings.
