# Supplementary material for: Bio-based poly(benzimidazole-co-amide)-derived N, O co-doped carbons as fast-charging anodes for lithium-ion batteries
Source: Nanoscale Adv. 2024 Aug 27;6(20):5181–92. doi: 10.1039/d4na00416g (PMC11376043; doi:10.1039/d4na00416g)
Supplement: NA-006-D4NA00416G-s001 [file NA-006-D4NA00416G-s001.pdf]

Supplementary Information (SI) for Nanoscale Advances.
This journal is © The Royal Society of Chemistry 2024

# Bio-based poly(benzimidazole-co-amide) as a fast charging anode of lithium-ion battery

Kottisa Sumala Patnaik[a], Bharat Srimitra Mantripragada[a], Rajashekar Badam[a], Koichi Higashimine[b], Xianzhu Zhong[a], Tatsuo Kaneko[a] and Noriyoshi Matsumi[a,c,d*]

[a]Graduate School of Advanced Science and Technology, Japan Advanced Institute of Science and Technology (JAIST), Nomi, Ishikawa 923-1292, Japan.

E-mail: matsumi@jaist.ac.jp

[b] Centre for Nano Materials and Technology, Japan Advanced Institute of Science and Technology (JAIST), Nomi, Ishikawa 923-1292, Japan

[c] Division of Transdisciplinary Sciences, Japan Advanced Institute of Science and Technology (JAIST), Nomi, Ishikawa 923-1292, Japan

[d] Elements Strategy Initiative for Catalysts and Batteries (ESCIB), Kyoto University, Nishikyo-ku, Kyoto 615-8245, Japan.

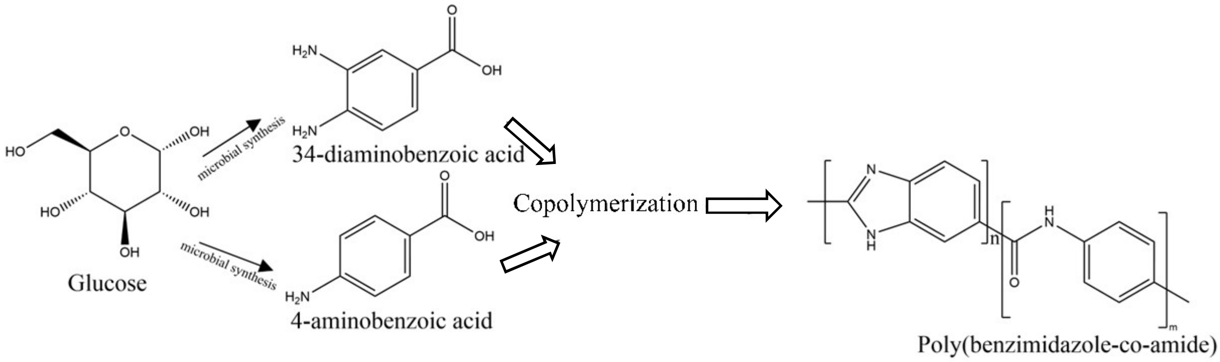


*Synthetic scheme1: Synthetic procedure for poly(benzimidazole-co-amide) [1]*

## Calculation of R factor

- R factor is a measure of the number of carbon sheets arranged as single layers.

$$R = \frac{B}{A}$$

Where A refers to the background
B refers to the peak height

- The background A is determined by drawing a straight line connecting the data on either side of the peak. And B is determined by drawing a line tangent to the background estimate which intersects 002 in a single point.

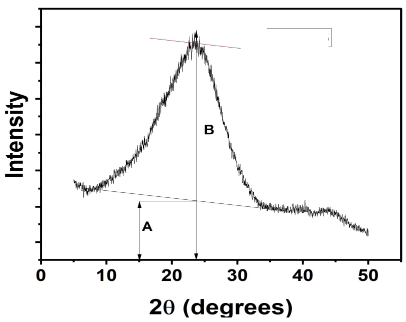


*Fig.S1: Procedure for determination of R factor*

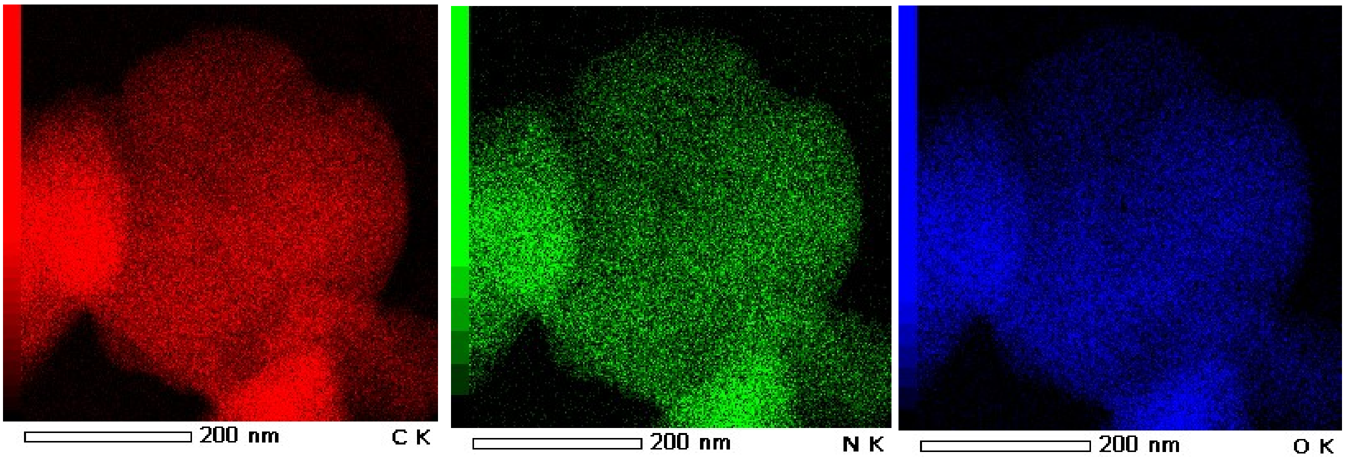

*Fig.S2: TEM-EDX elemental mapping of PYPBIPA8.5-1.5*

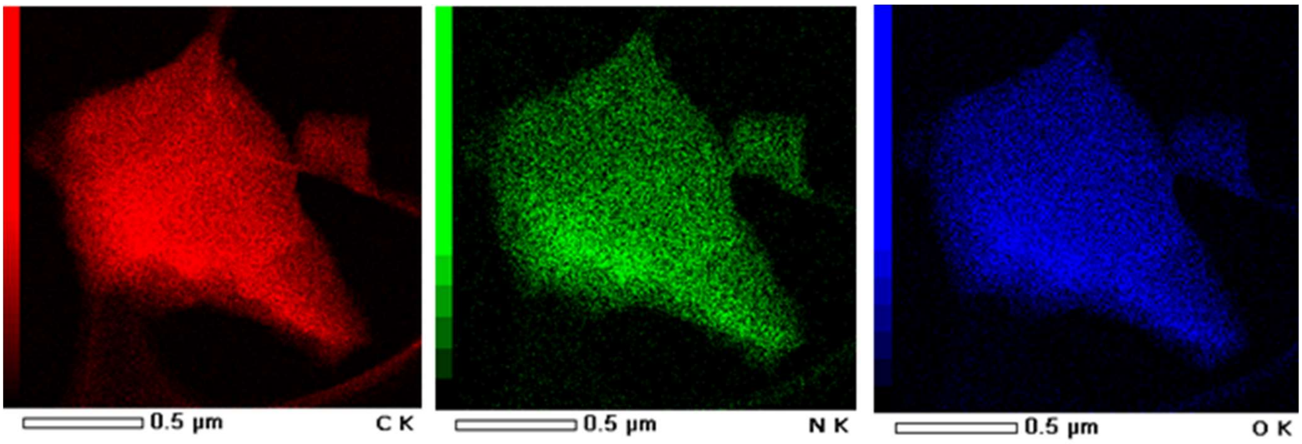

*Fig.S3: TEM-EDX elemental mapping of PYPBIPA7-3*

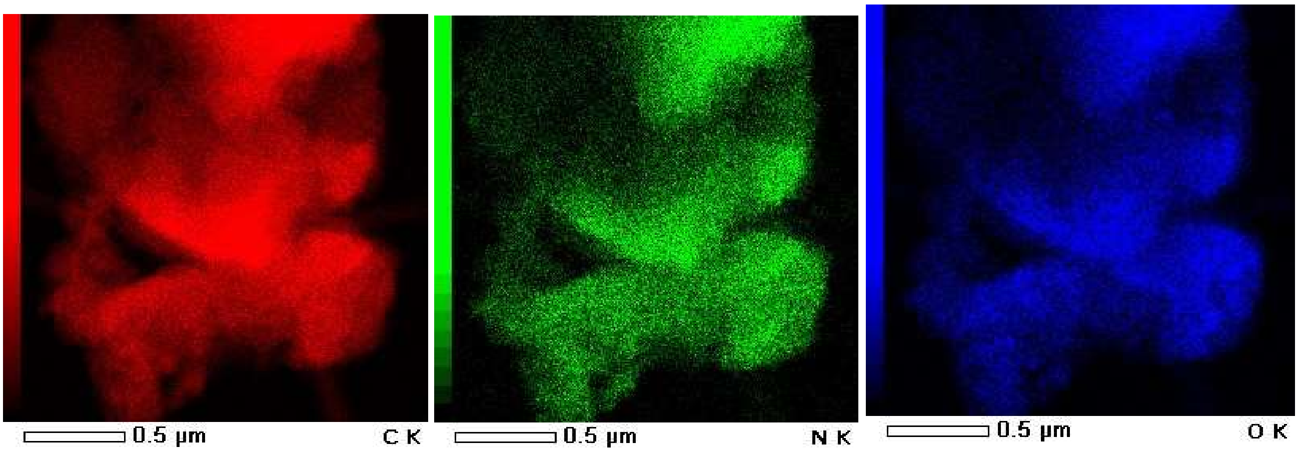

*Fig.S4: TEM-EDX elemental mapping of PYPBIPA5-5*

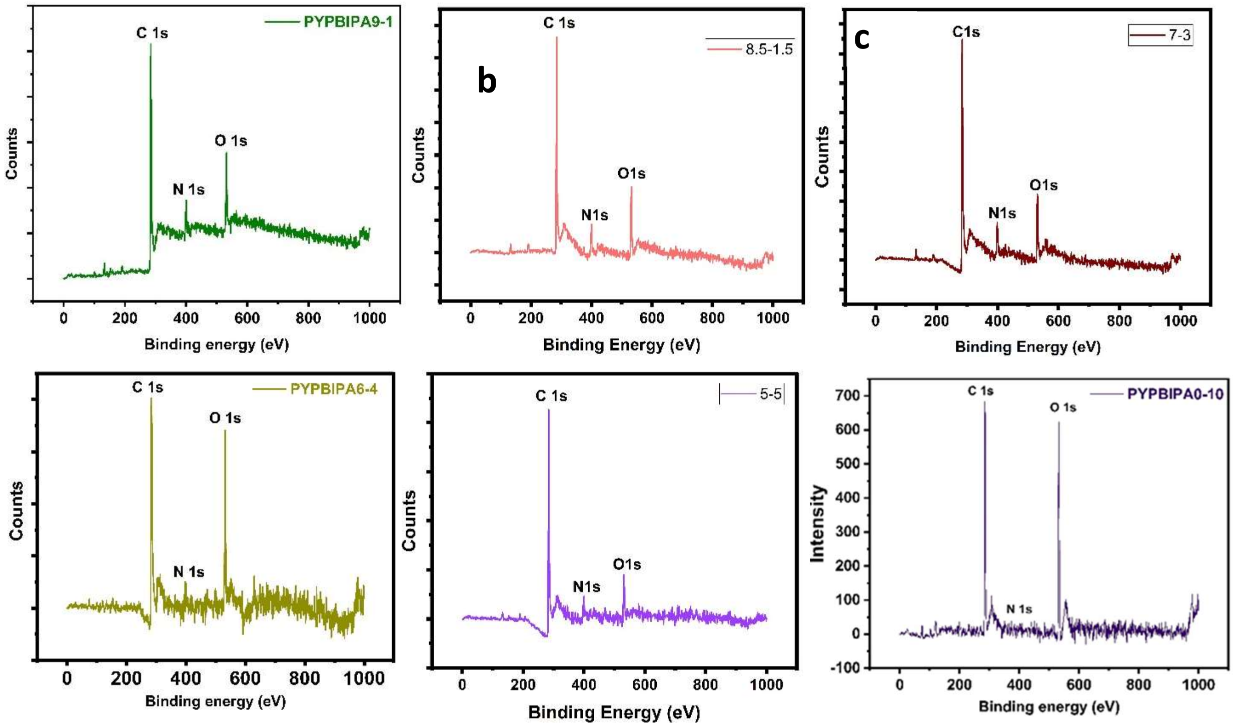


*Fig.S5: XPS survey spectrum of PYPBIPA(a)9-1 (b) 8.5-1.5 (c)7-3 (d) 6-4 (e) 5-5 (f) 0-10*

*Table S1: Nitrogen and oxygen doping contents in various carbon materials derived from PBIPA.*

| Carbon material | N at% | O at% |
|---|---|---|
| PYPBIPA10- 0[1] | 14.6 | 0.6 |
| PYPBIPA 9-1 | 12.1 | 11.8 |
| PYPBIPA8.5-1.5 | 11.2 | 13.1 |
| PYPBIPA7-3 | 9.9 | 20.0 |
| PYPBIPA6-4 | 9.2 | 21.8 |
| PYPBIPA5-5 | 8.0 | 25.0 |
| PYPBIPA0-10 | 2.6 | 31.3 |

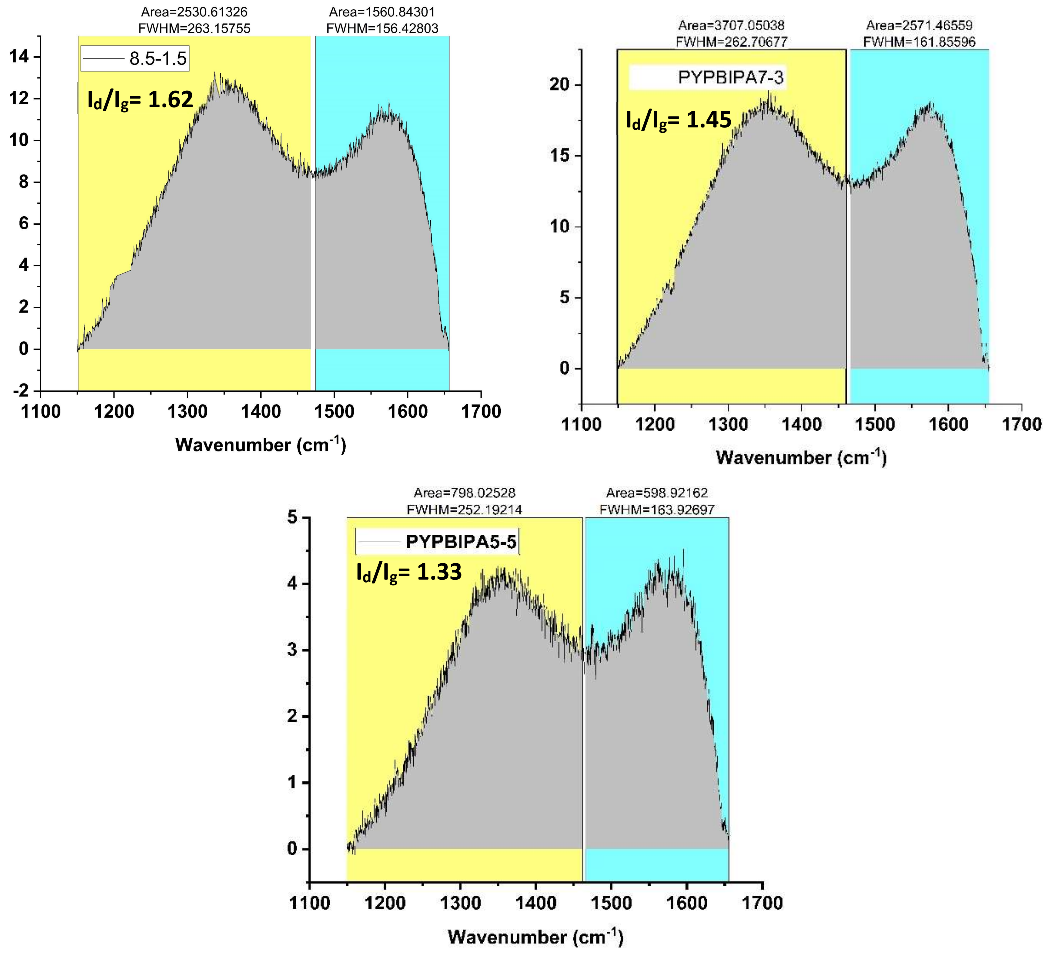


*Fig. S6: Ratio of the D peak to G peak based on the ratio of the area of D peak and G peak.*

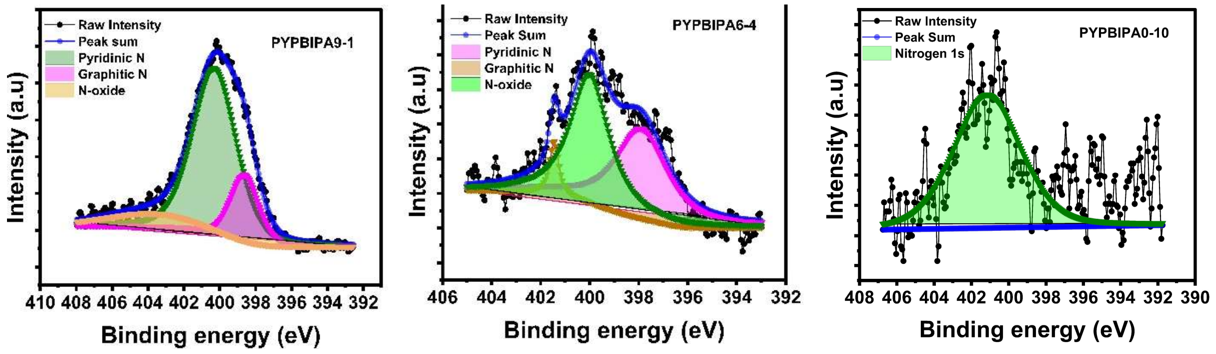


*Figure S7: Deconvoluted N1s spectra of (a) PYPBIPA9-1 (b) 6-4 and (c) 0-10*

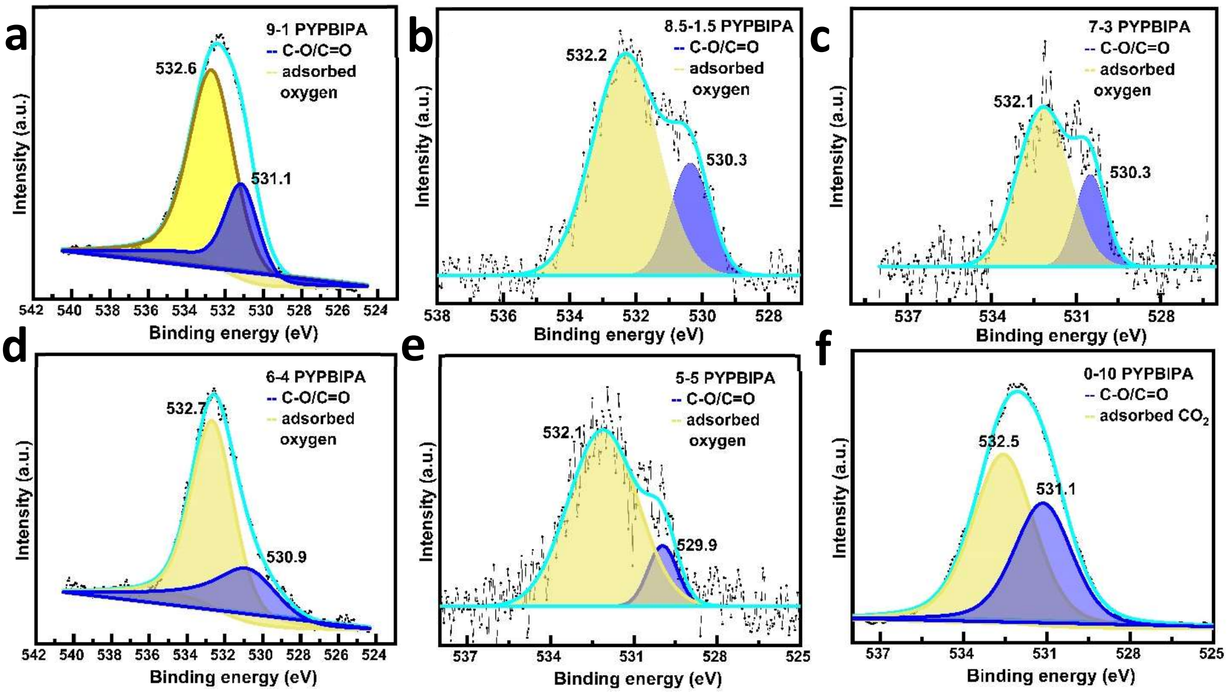


*Fig. S8: Deconvoluted O1s peak for PYPBIPA (a) 9-1 (b) 8.5-1.5 (c) 7-3 (d) 6-4 (e) 5-5 (f) 0-10*

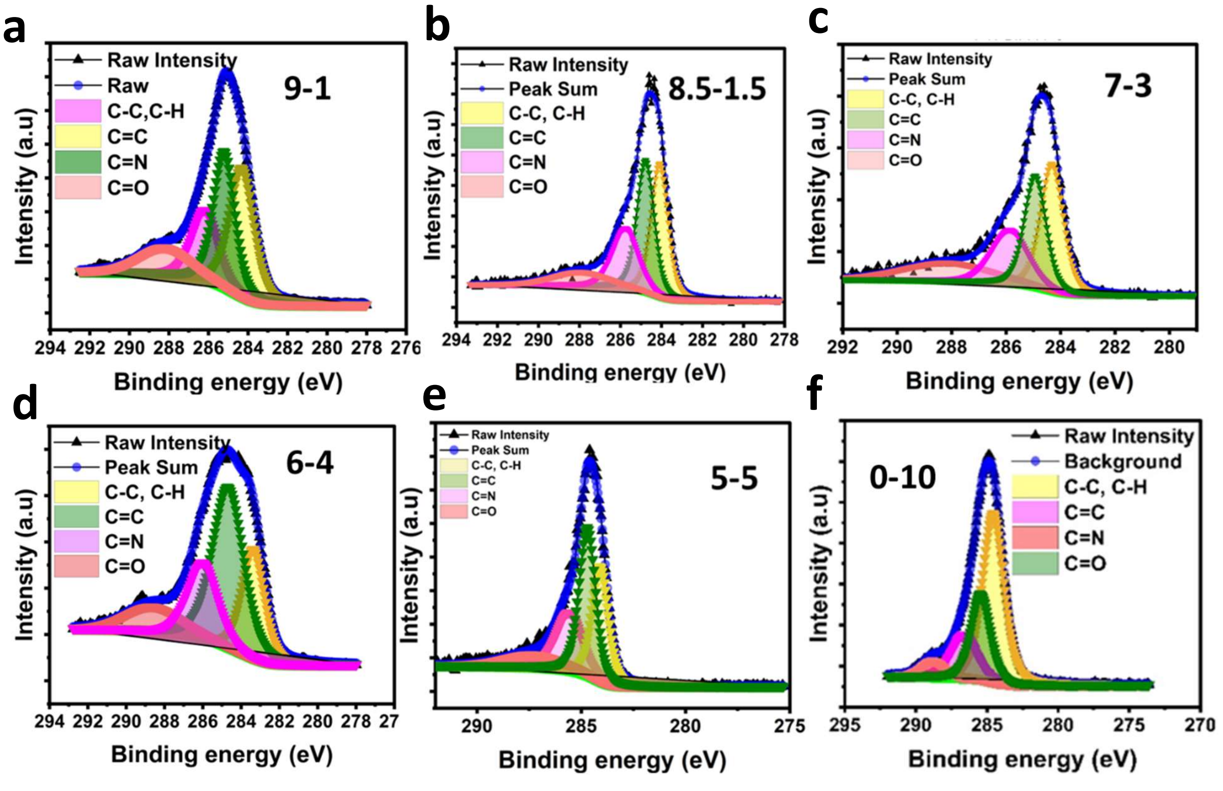


*Fig.S9: Deconvoluted C1s peak for PYPBIPA (a)9-1 (b)8.5-1.5 (c) 7-3 (d) 6-4 (e) 5-5 (f) 0-10*

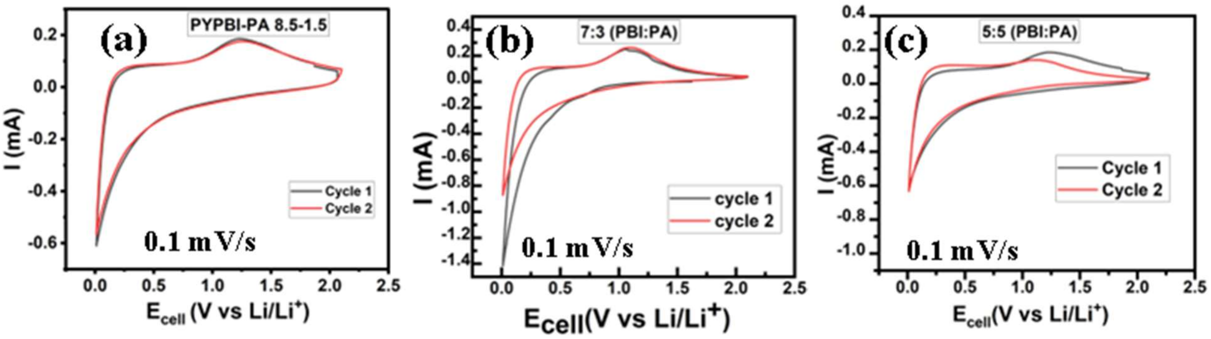


*Fig. S10: Cyclic voltammograms of PYPBIPA (a)8.5-1.5 (b) 7-3 and (c) 5-5*

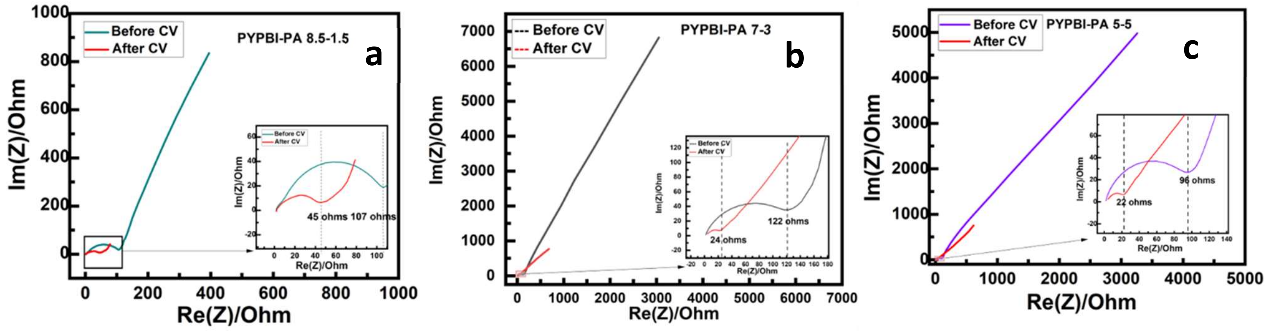


*Fig.S11: PEIS before and after cyclic voltammetry for PYPBIPA(a) 8.5-1.5 (b) 7-3 (c) 5-5*

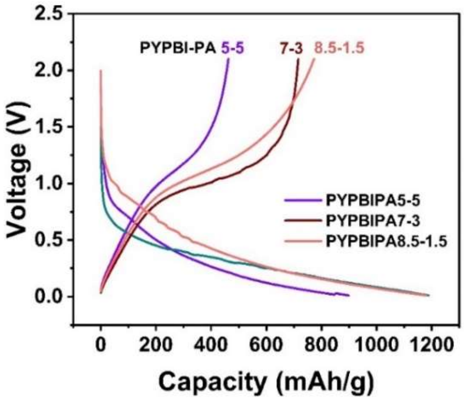


*Fig. S12: Initial charge-discharge plot for PYPBIPA8.5-1.5,7-3,5-5*

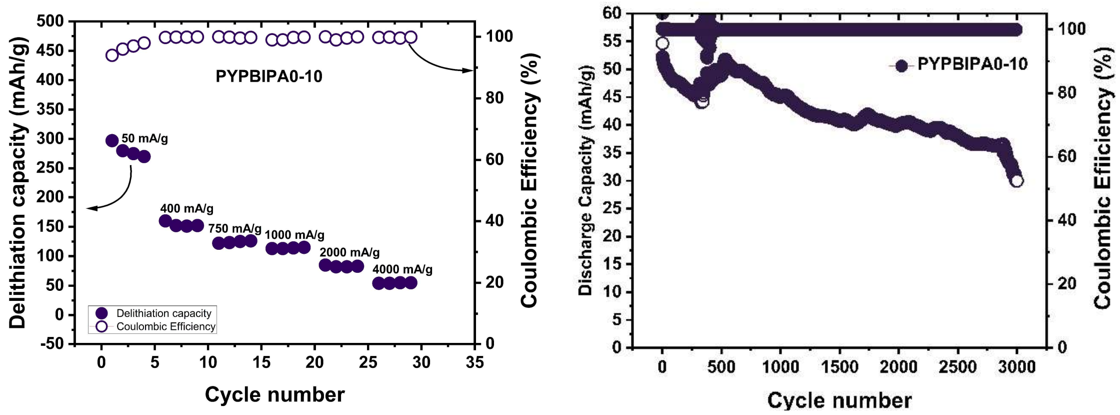


*Figure S13: (a) Rate studies (b) long cycling for PYPBIPA0-10*

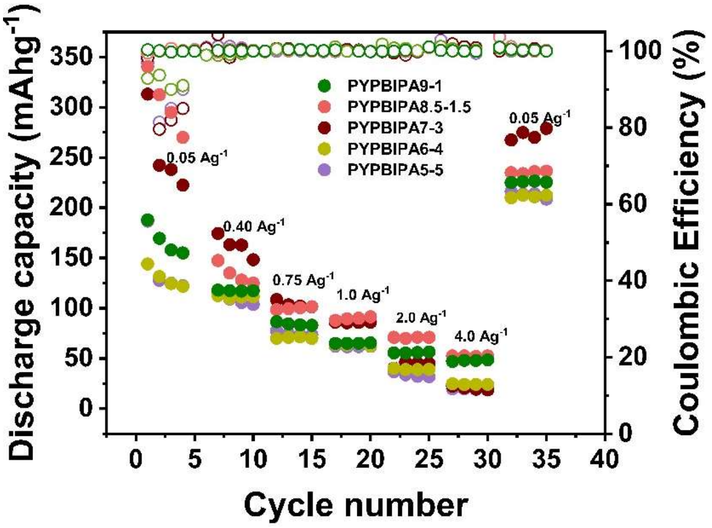


*Figure S14: Comparison of rate studies for PYPBIPA9-1, 8.5-1.5, 7-3, 6-4, and 5-5*

*Table S2: Comparison of rate studies of PYPBIPA10-0, 9-1, 8.5-1.5, 7-3, 6-4, 5-5 and 0-10*

| Current density (A/g) | | | Capacity (mAh/g) | | | | |
|---|---|---|---|---|---|---|---|
| | 10-0[1] | 9-1 | 8.5-1.5 | 7-3 | 6-4 | 5-5 | 0-10 |
| **0.05** | - | 330 | 600 | 500 | 250 | 270 | 280 |
| **0.40** | 206 | 235 | 256 | 325 | 220 | 211 | 152 |
| **0.75** | 168 | 167 | 199 | 204 | 140 | 147 | 125 |
| **1.0** | - | 130 | 180 | 171 | 128 | 123 | 113 |
| **2.0** | 125 | 111 | 140 | 90 | 80 | 64 | 85 |
| **4.0** | - | 94 | 100 | 38 | 48 | 39 | 55 |

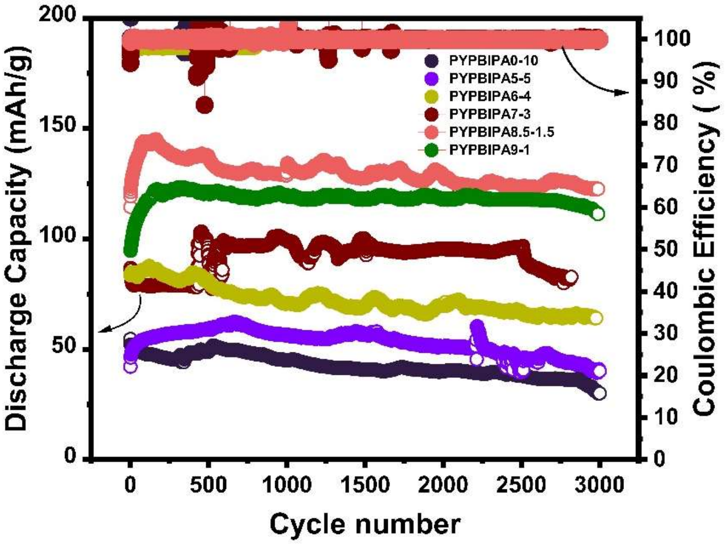


*Figure S15: Comparison of long cycling for PYPBIPA9-1, 8.5-1.5, 7-3, 6-4, 5-5 and 0-10*

*Table S3: Comparison of rate studies of PYPBIPA10-0, 9-1, 8.5-1.5, 7-3, 6-4, 5-5 and 0-10*

| Carbon material | Discharge capacity (mAh/g) | Capacity retention (%) |
|---|---|---|
| PYPBIPA 9-1 | 122 | 90 |
| PYPBIPA8.5-1.5 | 137 | 90 |
| PYPBIPA7-3 | 100 | 83 |
| PYPBIPA6-4 | 84 | 76 |
| PYPBIPA5-5 | 60 | 65 |
| PYPBIPA0-10 | 51 | 59 |

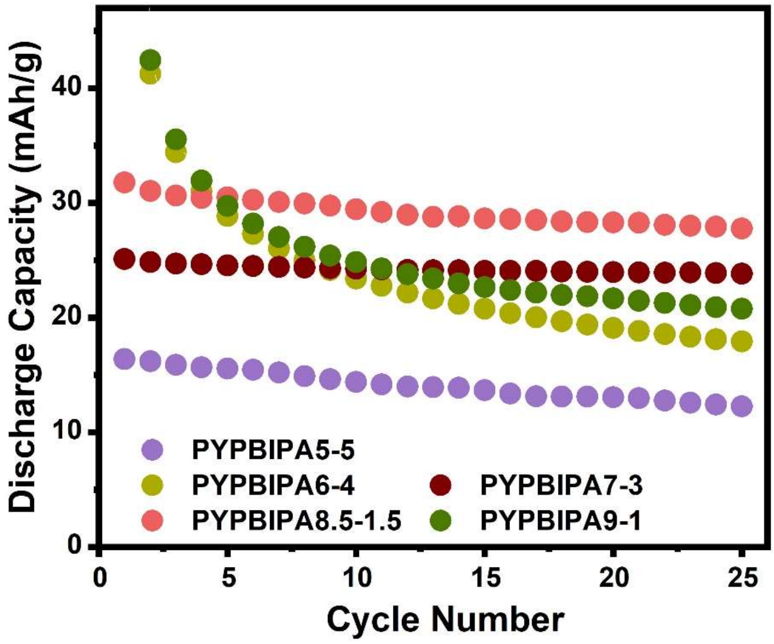


*Figure S16: Long cycling plot for symmetric cells of PYPBIPA9-1, 8.5-1.5, 7-3, 6-4, and 5-5.*

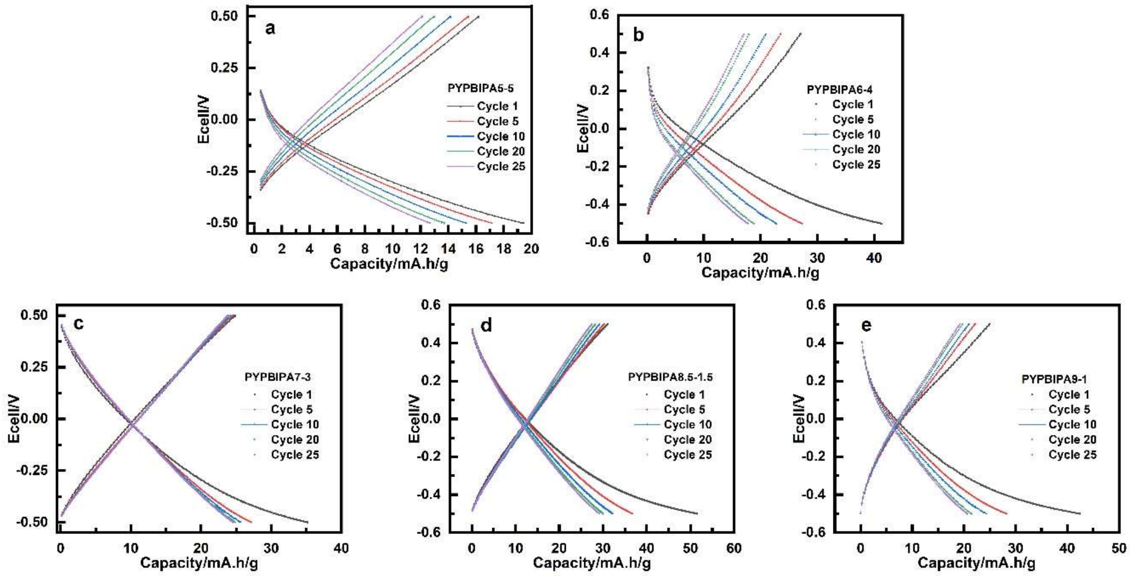


*Figure S17: Voltage vs capacity plots for PYPBIPA 0-10, 5-5, 6-4, 7-3, 8.5-1.5 and 9-1 symmetric cells*

*Table S4-Capacity retention of PYPBIPA materials after 25 cycles*

| Sample | Retention (%) | Cycles |
|---|---|---|
| PYPBIPA5-5 | 47.6% | 25 |
| PYPBIPA6-4 | 52.9% | 25 |
| PYPBIPA7-3 | 88.0% | 25 |
| PYPBIPA8.5-1.5 | 91.3% | 25 |
| PYPBIPA9-1 | 68.7% | 25 |

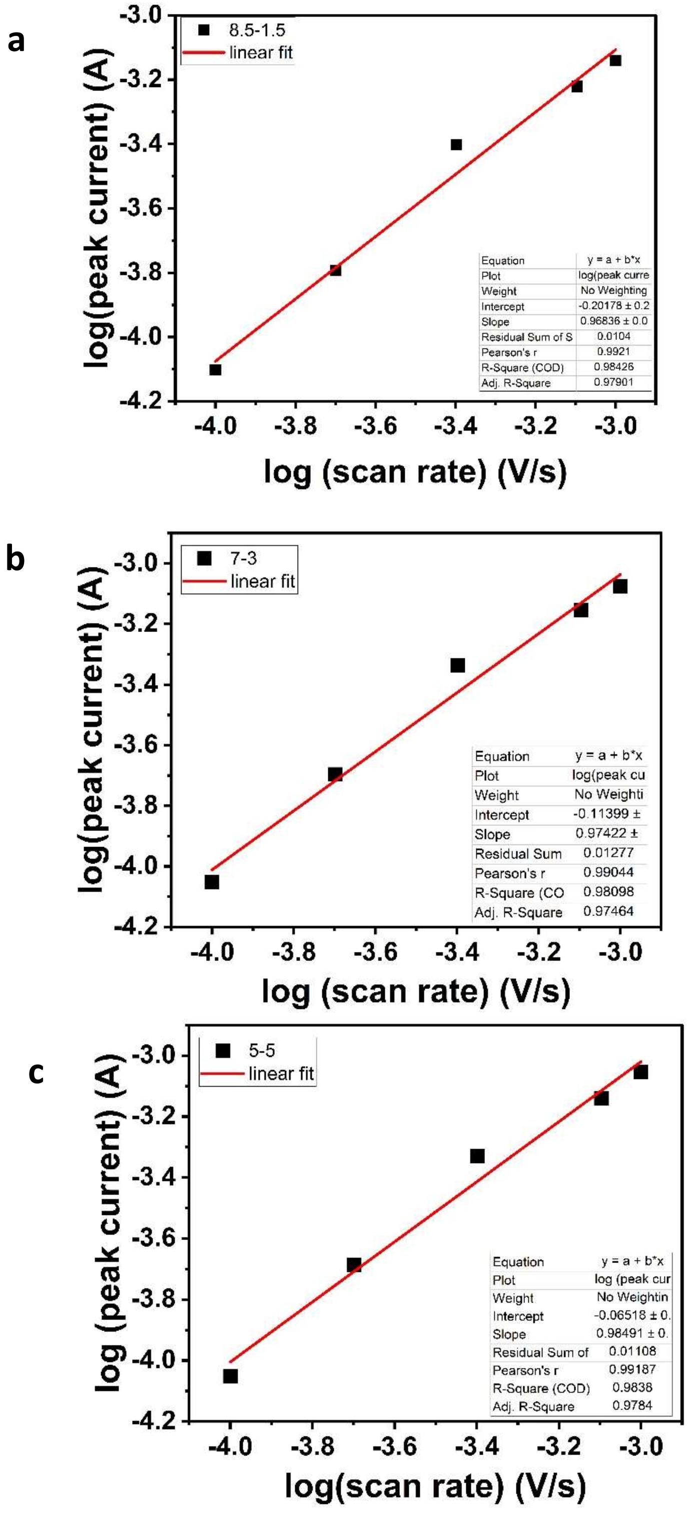


*Fig.S18: Linear fit of logi vs logv for PYPBIPA (a) PYPBIPA8.5-1.5 (b) 7-3 and (c) 5-5*

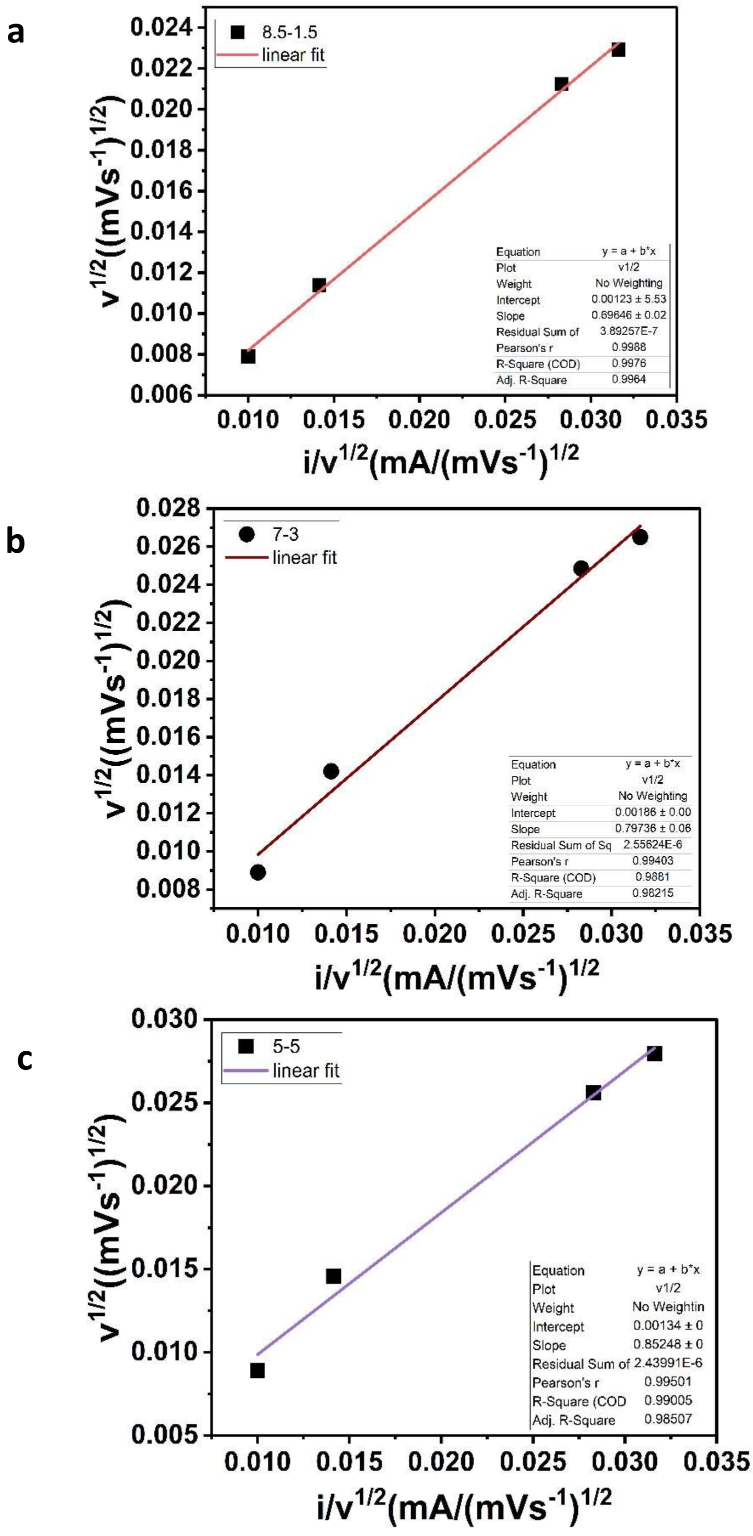


*Fig.S19: Linear fit of $i/v^{1/2}$ vs $v^{1/2}$ for PYPBIPA (a) PYPBIPA8.5-1.5 (b) 7-3 and (c) 5-5*

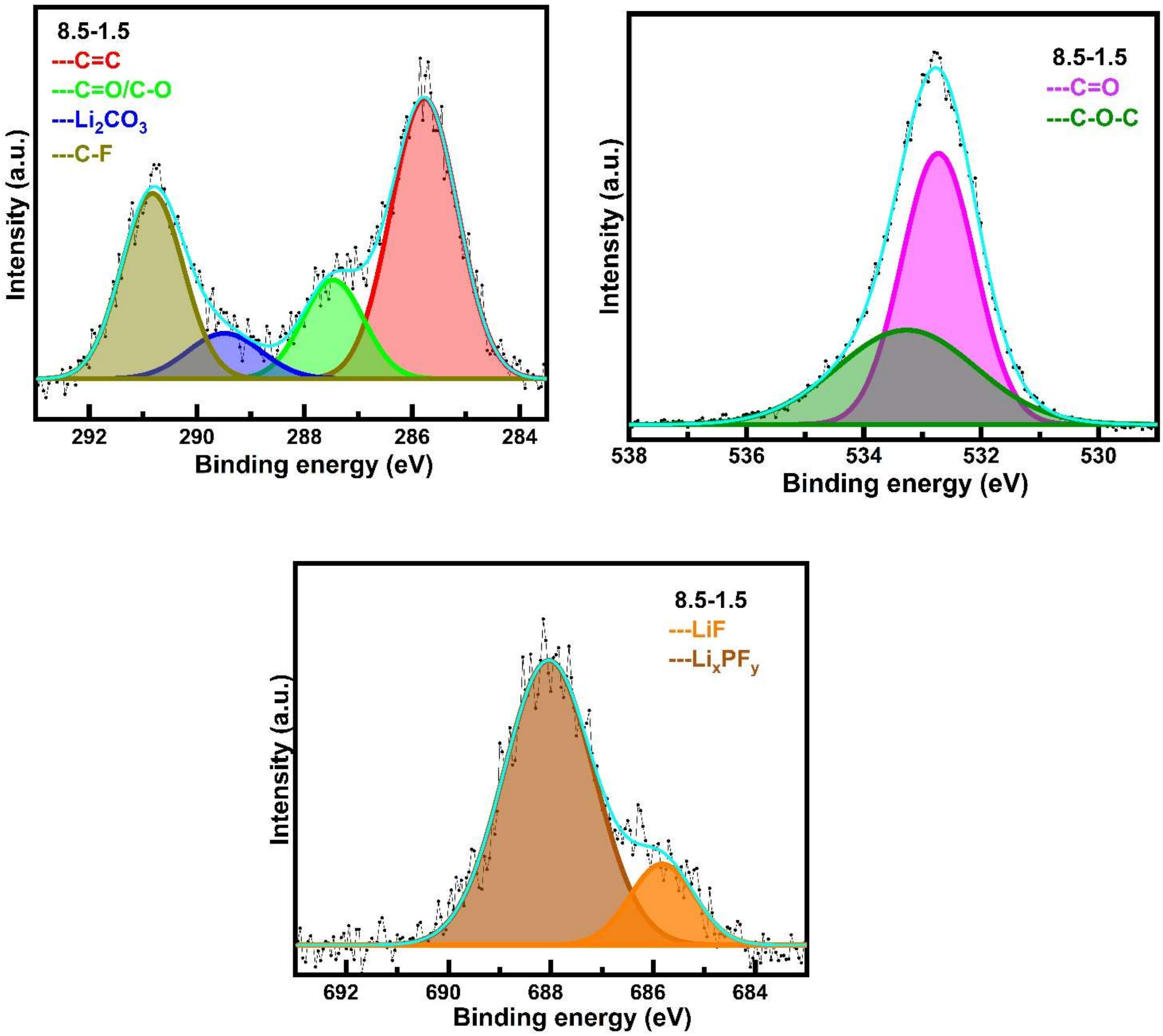


*Fig.S20: XPS of decrimped anode of PYPBIPA8.5-1.5 (a) C1s (b) O1s (c) F1s*

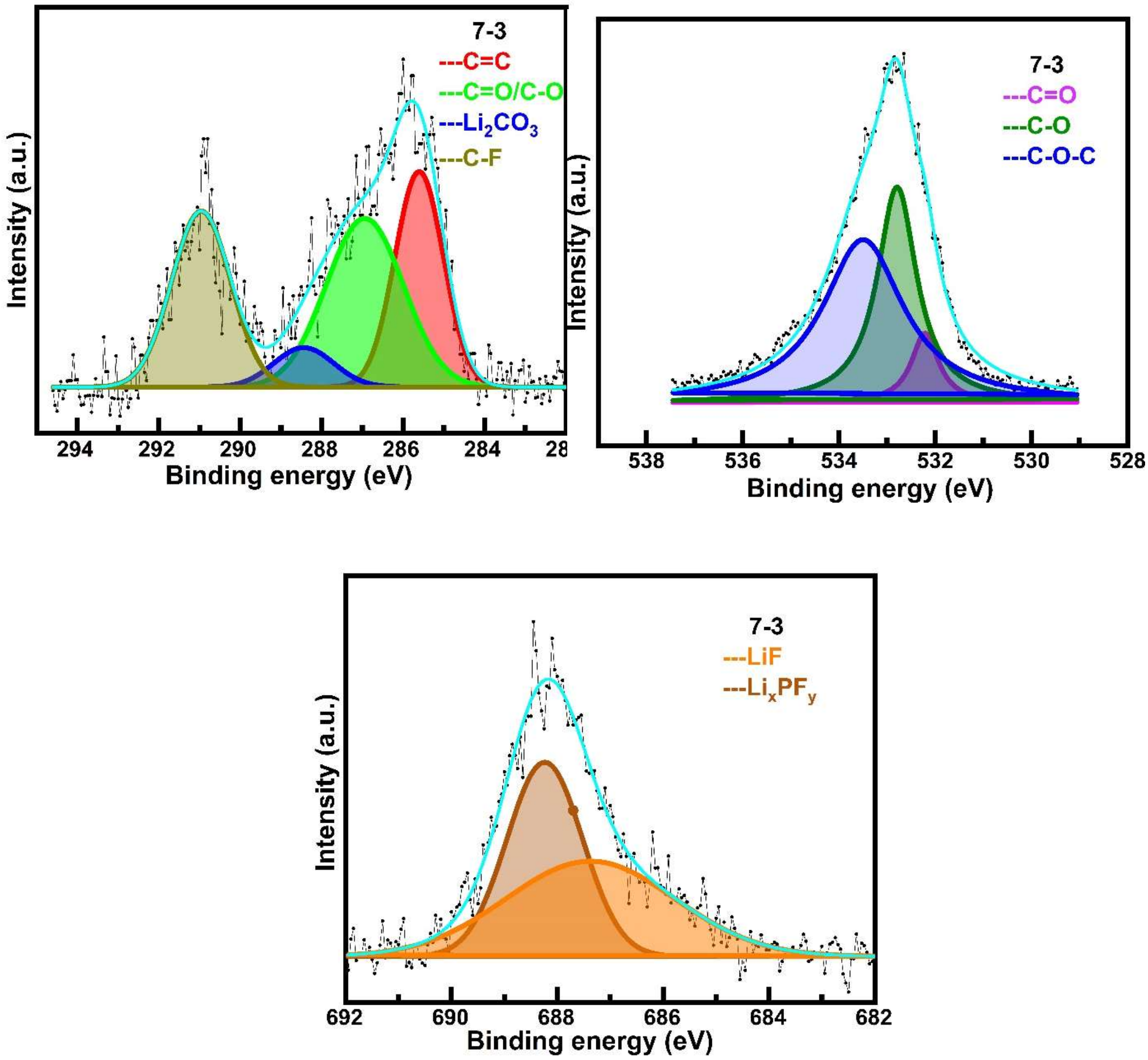


*Fig.S21: XPS of decrimped anode of PYPBIPA7-3 (a) C1s (b) O1s (c) F1s*

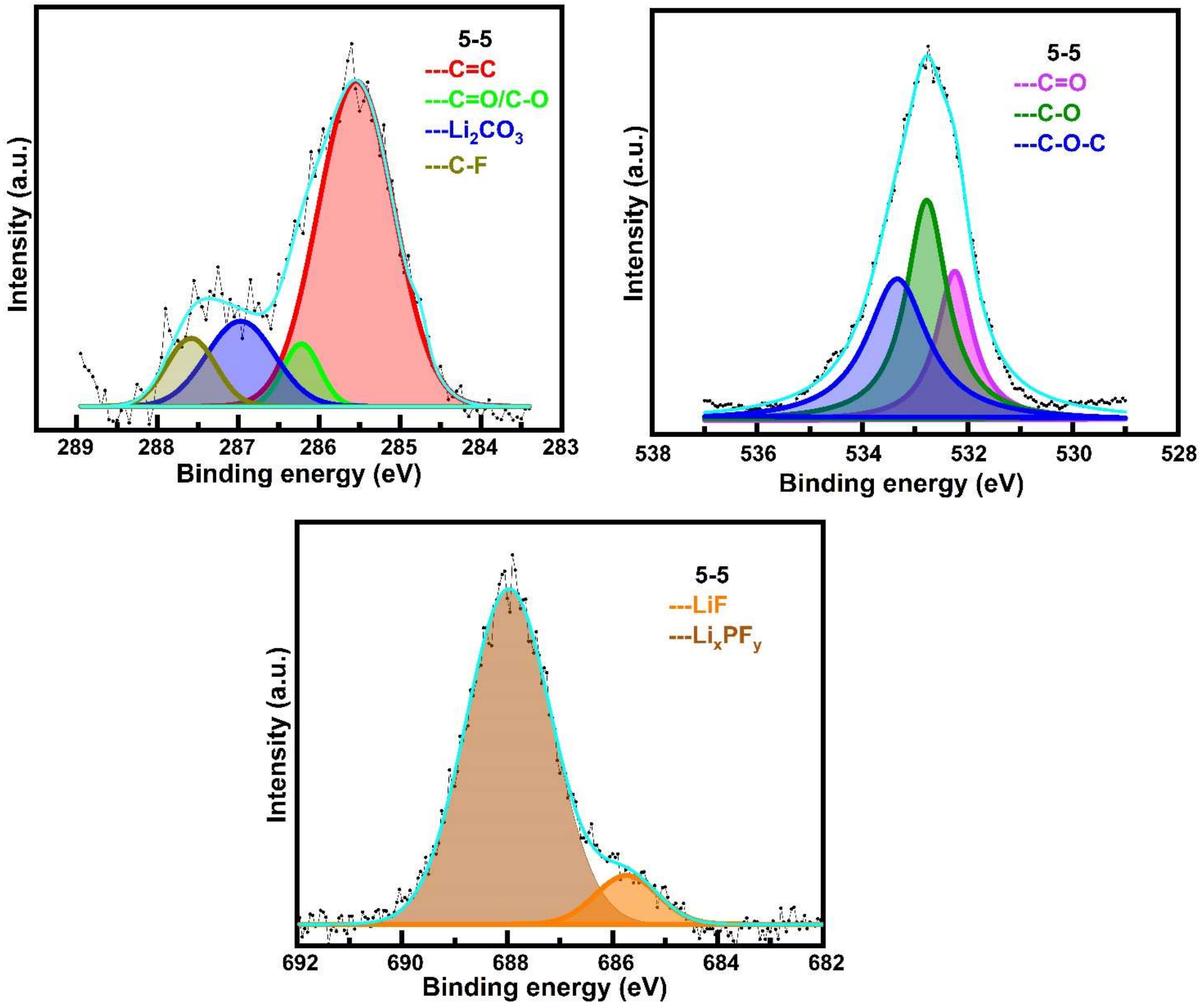


*Fig.S22: XPS of decrimped anode of PYPBIPA5-5 (a) C1s (b) O1s (c) F1s*

References:

[1] K. S. Patnaik, R. Badam, Y. Peng, K. Higashimine, T. Kaneko, N. Matsumi, *Chemical Communications* **2021**, *57*, 13704–13707.
